# Risk factors and causative organisms in microbial keratitis in daily disposable contact lens wear

**DOI:** 10.1371/journal.pone.0181343

**Published:** 2017-08-16

**Authors:** Fiona Stapleton, Thomas Naduvilath, Lisa Keay, Cherry Radford, John Dart, Katie Edwards, Nicole Carnt, Darwin Minassian, Brien Holden

**Affiliations:** 1 School of Optometry and Vision Science, University of New South Wales, Sydney, Australia; 2 Brien Holden Vision Institute, University of New South Wales, Sydney, Australia; 3 Vision CRC, University of New South Wales, Sydney, Australia; 4 The George Institute for Global Health, University of New South Wales, Sydney, Australia; 5 Moorfields Eye Hospital NHS Foundation Trust, London, United Kingdom; 6 The UCL Institute of Ophthalmology, London, United Kingdom; 7 IHBI, Queensland University of Technology, Brisbane, Australia; Xiamen University, CHINA

## Abstract

**Purpose:**

This study investigated independent risk factors and causative organisms in microbial keratitis in daily disposable contact lens (CL)-wearers.

**Methods:**

A multisite prospective case-control study was undertaken. Cases were daily disposable CL-wearers attending Moorfields Eye Hospital with microbial keratitis and those reported through a one-year surveillance study in Australia and in New Zealand. A population-based telephone survey identified daily disposable CL-wearing controls. Subjects completed a questionnaire describing CL-wear history, hygiene and demographics. The sample used for risk factor analysis was weighted in proportion to the CL-wearing population at each location. Corneal scrape results were accessed. Independent risk factors were determined using multiple binary logistic regression. Causative organisms in different CL-wear modalities were compared using a chi-squared test.

**Results:**

963 daily disposable CL-wearers were identified, from which 67 cases and 374 controls were sampled. Independent risk factors were; wearing CLs every day compared with less frequent use (OR 10.4x; 95% CI 2.9–56.4), any overnight wear (OR 1.8x; 95% CI 1.6–2.1), less frequent hand washing (OR 1.8x; 95% CI 1.6–2.0), and smoking (OR 1.3x; 95% CI 1.1–1.6). Certain daily disposable CLs (OR 0.2x; 95% CI 0.1–0.2) had protective effects. Environmental organisms were less frequently recovered with daily disposable CLs (20%), compared with other modalities (36%; p<0.02).

**Conclusion:**

Overnight wear, increased exposure in daily wear, smoking and poor hand hygiene are significant risk factors for microbial keratitis with daily disposable CLs. Risk varied with daily disposable CL type. The profile of causative organisms is consistent with less severe disease.

## Introduction

Daily disposable contact lenses were first introduced in the mid-1990s and between 2007–2011 comprised 24% of the total soft lens fittings internationally. [1] Given the increasing uptake of this modality, an understanding of the impact of these lenses on the risks of microbial keratitis is important.

Daily disposable lens wear eliminates the need for a contact lens storage case and lens care solutions. However, recent large scale epidemiological studies have indicated the incidence of microbial keratitis with daily disposable lenses is not lower than rates with planned replacement daily wear soft lenses, [2,3] however the relative risk varies with different daily disposable lens types. [3] The incidence of severe disease and disease causing vision loss is considerably reduced with daily disposable lenses, [2,3] which may be indicative of a different profile of causative organisms. [4]

Established risk factors for microbial keratitis with all daily wear lenses include overnight and increased days of wear, poor hand, lens and storage case hygiene, youth, male gender, smoking and Internet purchase. [2–5] Characteristics of wearers opting for daily disposable contact lens use might impose a different risk profile for daily disposable lens use compared to other lens wear modalities. The population wearing these lenses may be influenced by the convenience of the daily disposable modality, the increased cost and a tendency for practitioners to prescribe daily disposable lenses as problem solving lenses. There is also evidence for an ‘early adopter’ phenomenon where the wearer profile differs in the early period following introduction of new modalities. [6]

The aims of this study are to determine independent risk factors for all presumed and moderate/severe microbial keratitis in daily disposable lens wear in a large multicentre study, through a secondary analysis of existing studies where datasets were combined to allow detailed analysis of risk factors, and to describe causative organisms.

## Methods

### Selection of cases and controls

Cases were derived from a two year case control study at Moorfields Eye Hospital, London, UK commencing December, 2003 [3]^,^ S1 File and a one year national surveillance study in Australia and New Zealand commencing October, 2003. [2] S2 File Both studies identified new cases of contact lens related microbial keratitis in wearers using daily disposable and other soft contact lenses for the correction of refractive errors. Contact lens wearing control subjects were identified through population-based telephone surveys in all three countries during the study periods. Hospital presenting contact lens wearing controls from Moorfields Eye Hospital were also identified. Detailed descriptions of the definition and identification of cases and controls for each of the studies described here have been published elsewhere. [2,3,7–9] Ethics Committee approval was obtained in each of the regions prospectively (two central Human Research Ethics Committees, London and University of NSW, and 63 regional centres in Australia and New Zealand) and the study approvals were ratified before the commencement of the study by the University of New South Wales Human Research Ethics Committee. Cases provided written consent to allow clinical data to be accessed, while controls consented orally due to the benign nature of the survey, which was the only data collected, and the scale of the study. These consent procedures were approved by all Ethics Committees involved in the study.

### Disease definition and severity criteria

Microbial keratitis was defined by either a positive corneal culture or a corneal infiltrate and overlying epithelial defect with one or more of the following features: (i) any part of the lesion being within the central 4 mm of the cornea, (ii) uveitis, or (iii) pain as reported by the wearer or practitioner. All cases presented with a corneal infiltrate. Cases were classified using a previously described [2,3] and validated [10] severity grading scheme, as severe if they resulted in visual loss of equal to, or more than, two lines of best corrected spectacle acuity compared with the pre-event visual acuity, or 6/6 where pre-event acuity was not available, with no other attributable cause. Cases without vision loss were classified as moderate if they had one or more of the following features: (i) a positive corneal culture, (ii) any part of the lesion being within the central 4mm of the cornea, (iii) hypopyon or (iv) four or more hospital visits. Other cases of microbial keratitis were classified as mild.

### Possible risk factors

Self-administered (cases) or telephone-administered (controls) questionnaires were used to identify potential risk factors for disease. Risk factors considered for analysis included;

1. Demographic factors. Age group (≤25 years, 26–50 years or more than 51 years), gender and socioeconomic class.
2. Lens wear history factors. Indication for lens wear (myopia, astigmatism, hypermetropia or presbyopia); duration of current lens wear (≤6 months or more than 6 months); lens wear modality (strict daily wear only, any occasional overnight wear, defined as less often than one night per week and extended wear defined as one night per week or more often); lens wear frequency (occasional defined as less than 1 day/week, part time as 1–5 days per week and full-time as 6–7 days per week; lens material (Etafilcon A, Nelfilcon A, Hilafilcon B or other); reuse of daily disposable lenses (yes or no); lens age; period since last aftercare (within the last 12 months or longer than 12 months); Internet purchase (always/sometimes or never).
3. Other behaviors. Handwashing prior to handling lenses (Yes or no/not always); smoking; swimming (none, with lenses or without lenses); showering while wearing lenses.

### Sampling and statistical methods

Nine hundred and sixty three daily disposable lens users were identified in the two studies. The demographics of all daily disposable wearers (n = 963) were compared with users of other lens modalities (n = 3078) using chi-squared analysis.

For the risk factor analysis, cases and control wearers using daily disposable lenses only were considered. To best represent the combined risk factors across all three locations, the sample of cases and controls was weighted in proportion to the contact lens wear penetrance in these three countries. The contact lens wear penetrance [9,11] was converted to point estimates of wearers aged between 15 and 64 years. Based on the size of the contact lens wearing population in each country, the proportion of daily disposable lens wearers was in the ratio 25:5:70 in Australia, New Zealand and UK respectively. This ratio was obtained by applying sample weights of 1, 0.55 and 0.38 to the Australia, New Zealand and UK samples respectively.

Univariate analysis of the potential risk factors for all severities of keratitis and for moderate/severe microbial keratitis in daily disposable contact lens use was conducted initially using chi-squared analysis (S3 File). Any factors significant at *p*<0.2 were considered for multivariate analysis using stepwise (backward elimination followed by forward entry) logistic regression. The final model included only factors significant at *p*<0.05. Odds ratios and 95% confidence intervals were used to summarize significant findings. Sample weights and clustering effects of study location was accounted in the weighted logistic model. For comparison purposes, an unweighted full dataset logistic model was also attempted using similar modelling methods. The Hosmer-Lemeshow test was used to show the goodness of fit and area under the receiver operating characteristic curve determined the discriminatory ability of the models. Univariate population attributable risk percentage (PAR %) were calculated to estimate the proportion of total cases that would be reduced by removing the risk factor from the population. PAR % is the rate of occurrence of the condition that can be attributed to the risk factor. Combined PAR % for two risk factors was estimated as follows:

Combined PAR % = {1−(1−PAR[risk factor 1])*(1−PAR[risk factor 2])} × 100

The statistical analysis was conducted using SPSS Version 18.0 (SPSS Inc, IL) and STATA 10.0 (STATA Corporation TX).

### Corneal scrapes

Corneal scrape results for 255 cases comprising 55 daily disposable wearers and 200 wearers of non-daily disposable soft and silicone hydrogel lens modalities, were collected from the three geographic sites. Cases were reported from multiple settings, and the criteria for a positive scrape were subject to the individual laboratory and practitioner criteria and their interpretation of the test result. If the laboratory report was available the following criteria was applied: culture proven cases were defined as cases where an organism was identified on more than one medium or on one solid medium with organisms having the same morphology as organisms visualized in the corneal scrape. If the organism was recovered from one medium only and/or after long periods of incubation, the result was considered negative. Scrape results were categorized and a chi-square test was used to compare the frequency of recovery of different organisms between daily disposable and other lens wearers.

## Results

Demographics (age and occupation) are shown for all daily disposable contact lens wearers (Table 1). Compared with other lens modalities, a higher proportion of daily disposable users were in the middle-aged group (*p*<0.01), were male (*p*<0.01) and were in professional/managerial jobs (*p*<0.01).

**Table 1:** Demographic variables for wearers of daily disposable and other lens wear modalities.

Demographic Variable	Category	DD* n (%)	Strict DW^†^ n (%)	Any Occ ON Wear^‡^ n (%)	EW^§^ n (%)	Total
Age group	Younger (< = 25)	169 (17.7%)	361 (25.5%)	137 (31.0%)	57 (21.8%)	724 (23.5%)
	Middle-Aged (25–50)	697 (72.8%)	896 (63.2%)	274 (62.0%)	172 (65.6%)	2039 (66.2%)
	Older (>50)	91 (9.5%)	160 (11.3%)	31 (7.0%)	33 (12.6%)	315 (10.2%)
Gender	Male	380 (39.6%)	462 (32.1%)	172 (38.4%)	112 (42.3%)	1126 (36.2%)
	Female	579 (60.4%)	977 (67.9%)	276 (61.6%)	153 (57.7%)	1985 (63.8%)
Occupation	Tier 1 (Managers, professionals, technical occupations)	642 (75.0%)	747 (67.1%)	237 (69.3%)	130 (68.1%)	1756 (70.2%)
	Tier 2 (Administrative, skilled trades, personal services)	188 (22.0%)	285 (25.6%)	73 (21.3%)	49 (25.7%)	595 (23.8%)
	Tier 3 (Sales/customer service, process/machine operators, elementary professions)	26 (3.0%)	81 (7.3%)	32 (9.4%)	12 (6.3%)	151 (6.0%)

*DD = Daily disposable contact lens wearers

^**†**^Strict DW = Strict daily wear lens use only, no overnight wear

^‡^Any Occ ON Wear = Any occasional overnight wear of lenses—less often than once per week

^§^EW = Extended wear of lenses—one night a week or more often

The full sample of daily disposable contact lens users comprised 166 cases of microbial keratitis (78 moderate/severe) and 797 controls (Table 2). After sample weights of 100%, 55% and 38% for Australia, New Zealand and UK respectively were applied to the complete dataset, the weighted sample (n = 441), comprising 67 cases of microbial keratitis (32 moderate/severe) and 374 controls was used for univariate and multivariable analysis.The univariate analysis is presented as supplementary data. S3 file.

**Table 2:** Microbial keratitis cases and controls using daily disposable contact lenses by location.

Region	All Microbial Keratitis	Moderate/Severe Microbial Keratitis	Controls
UK	160	75	653
Australia	6	3	104
New Zealand	0	0	40
Total (n = 963)	166	78	797

The distribution of wear modality (strict daily wear, occasional overnight wear and extended wear) differed between the daily disposable and frequent replacement soft lens users (*p*<0.001). Strict daily wear was reported in 313/440 (71%) of daily disposable and 361/555 (65%) of frequent replacement soft lens users. Occasional overnight use and extended wear was reported by 117/440 (27%) and 10/440 (2%) of daily disposable and 137/555 (25%) and 57/555 (10%) of frequent replacement soft contact lens users respectively.

### Univariate analysis

Smoking (*p* = 0.016), hand washing (*p*<0.001), swimming (*p* = 0.007), showering (*p* = 0.02), lens wear modality (*p<0*.*001*) and frequency (*p*<0.001) and lens disposable lens type (*p* = 0.005) were considered for the multivariate analysis. Risk factors which did not reach significance included age (p = 0.7), male gender (*p* = 0.3), duration of lens wear (*p* = 0.6), reason for wear (*p* = 0.9), reuse of daily disposable lenses (*p* = 0.9), time since aftercare (p = 0.6) and Internet purchase (*p* = 0.9). Factors significant for all presumed cases, were also considered for the multivariate analysis of moderate/severe cases.

### Multivariate analysis

Independent risk factors identified for all microbial keratitis and for moderate/severe disease are shown in Table 3, ranked according to PAR %. The combined PAR % for wearing lenses every day and failing to consistently wash hands prior to handling lenses was 92% for all keratitis and 87% for moderate/severe disease.

**Table 3:** Independent risk factors for all and moderate/severe microbial keratitis identified by multiple logistic regression analysis.

	All Presumed Microbial Keratitis				Moderate/Severe Microbial Keratitis			
Risk factor	Odds Ratio	p-value	95% C*	PAR %^†^	Odds Ratio	p-value	95% CI	PAR %
**Wear frequency**								
Occasionally	**Referent**				**Referent (Occasionally/Part time)**			
Part-Time	2.83	0.176	0.6–12.8	NS‡				
Everyday	10.41	***0*.*007***	1.9–56.4	85%	6.28	***<0*.*001***	3.1–12.9	72%
**Hand washing**								
Yes	**Referent**			34%	**Referent**			45%
No/Not always	1.79	***<0*.*001***	1.6–2.0		2.41	***<0*.*001***	2.0–2.9	
**Lens material type**								
Etafilcon A	**Referent**				**Referent**			
Nelfilcon A	3.98	***<0*.*001***	3.6–4.4	36%	4.83	***<0*.*001***	2.5–9.4	46%
Hilafilcon B	2.06	***<0*.*001***	1.5–2.8	7%	2.61	***<0*.*001***	2.1–3.3	13%
Others	2.52	***<0*.*001***	2.3–2.8	21%	2.80	***<0*.*001***	1.7–4.6	31%
**Mode of wear**								
Strict DW	**Referent**			28%	**Referent**			27%
Any Occ ON Wear	1.83	***<0*.*001***	1.6–2.1		1.69	***0*.*034***	1.04–2.7	
**Smoking**								
Non-Smoker	**Referent**			16%				
Smoker	1.29	***0*.*016***	1.1–1.6				NS^c^	

Bold italicised values are those significant at p<0.05

*95% confidence intervals

^†^Population attributable risk percentage

^‡^Not significant

In the model for all microbial keratitis, the area under the receiver operating curve was 77% indicating good ability of the model to discriminate between cases and controls. The Hosmer-Lemeshow test for goodness of fit indicated acceptable fit (p = 0.2). Similarly the moderate/severe model showed good discriminatory ability (79%) and the Hosmer-Lemeshow test indicated an acceptable fit (*p* = 0.7). When the logistic model was developed without the use of sample weights, the factors that were significant in the weighted model remained significant in the unweighted model. However, age was an additional factor in the unweighted model, where age group 26–50 years had a higher risk of any microbial keratitis (OR: 1.8 95% CI: 1.6–1.9) compared to those aged < = 25 years. It is noted that age was significant only at the 10% level in the univariate analysis of unweighted data (p = 0.101).

### Microbial analysis

Corneal scrape results were available for 55 daily disposable wearers and 200 wearers of other modalities and results are shown by modality in Table 4. Environmental organisms (*p* = 0.02) including Gram negative bacteria (*p*<0.05) were less frequently recovered from daily disposable infections compared with those from other lens modalities. *Acanthamoeba* was not isolated from any of the daily disposable wearers.

**Table 4:** Corneal scrape results for daily disposable wearers and wearers of other soft and silicone hydrogel lenses from three geographic sites.

Scrape Result	Daily Disposable Cases (n = 55)	Non-Daily Disposable Soft and Silicone Hydrogel Cases (n = 200)	P
Culture negative	35 (64%)	99 (50%)	0.063
Gram positive bacteria*	9 (16%)	27 (14%)	NS^†^
*Staphylococcus aureus*	0	4 (2%)	
Coagulase negative staphylococci/other staphylococci	8 (15%)	15 (8%)	
*Streptococcus pneumoniae*	0	1 (1%)	
*Streptococcus viridans*	1 (2%)	1 (1%)	
*Corynebacterium sp*.	0	3 (2%)	
Other Gram positive bacteria	0	3 (2%)	
Environmental organisms^‡^	11 (20%)	74 (37%)	***0*.*018***
Gram negative bacteria	10 (18%)	64 (32%)	0.046
*Pseudomonas aeruginosa* or *spp*.	9 (6%)	53^§^ (27%)	
*Serratia marcescens*	1 (2%)	8 (4%)	
*Klebsiella oxygenate*	0	2 (1%)	
Other Gram negative bacteria	0	1 (1%)	
*Nocardia* spp.	0	2 (1%)	NS
Acanthamoeba	0	5 (3%)	NS
Fungi	1 (2%)	3 (2%)	NS
*Fusarium dimerum*	1 (2%)	0	
*Acremonium* sp.	0	1 (1%)	
*Trichosporon mucoides*	0	1 (1%)	
*Candida* sp.	0	1 (1%)	

Bold italicised values are those significant at p<0.05

*Gram positive bacteria included endogenous species and excluded *Nocardia* spp.

^**†**^NS = not significant

^‡^Environmental organisms included Gram negative bacteria, fungi, *Acanthamoeba* spp. and *Nocardia* spp.

^§^Included 5 polymicrobial *Pseudomonas aeruginosa* cultures

## Discussion

This study is the first to report risk factors for all severities of microbial keratitis and for more severe disease in daily disposable lens wearers only. The study involved data collected through surveillance studies in Australia and in New Zealand and a case control study in London, UK. The study sites used identical protocols for diagnosing and classifying contact lens related microbial keratitis and compliance questionnaires for establishing risk factors. Risk factor data for all lens modalities has been previously published from the UK site [3] and from Australia, [2] however this is the first report of risk factors associated with daily disposable contact lenses and of causative organisms in this modality.

A weighted sample of cases and controls in different geographic locations was analysed to improve generalizability of these results and account for geographic factors, such as climate. While environmental microorganisms comprise the largest group of causative organisms for microbial keratitis, such organisms are less frequently recovered in daily disposable contact lens wearers.

Independent risk factors for all presumed microbial keratitis in daily disposable wearers included increased exposure to lens wear, both in terms of more days per week of lens wear and occasional overnight lens wear. Using lenses more frequently was associated with the largest population attributable risk of 93% for all presumed cases and 72% for more severe cases. This dose dependent effect has been reported previously for all lens modalities, [3,5] and would not be unexpected given daily disposable lenses interact with the ocular surface in a similar fashion to other modalities. Occasional overnight wear was a significant factor in both univariate and multivariate analysis for moderate/severe cases. There were no moderate/severe cases in extended wear, and only six controls who wore daily disposable lenses on an extended wear basis in the dataset. The small sample size limits our ability to explore this relationship.

This study confirms that washing hands remains an important hygiene measure even with daily disposal of lenses. Consistently washing hands prior to lens handling decreases the population attributable risk by almost 50% for moderate/severe microbial keratitis. Wearing lenses every day and failing to wash hands was associated with 90% of the disease load. These risk factors suggest inoculation of the organisms to the ocular surface and increased retention time due to the presence of the lens are key in the pathogenesis of contact lens related microbial keratitis, which is consistent with animal models of lens wear and infection. [12,13]

It is unclear why certain daily disposable lens types are protective for the risk of infection. A previous analysis of non-ulcerative complications indicated that certain daily disposable contact lenses had a 2-fold increase in mechanical complications, such as corneal abrasions, compared to other frequent replacement lenses. [14] Other studies have shown daily disposable wearers may have more difficulty handling lenses than frequent replacement wearers [15] and some types of daily disposable lenses are more difficult to remove than others, [16] which may increase the risk of mechanical complications. Since this study was completed, the design and material of several of the daily disposable lens brands has been modified and it may not possible to extrapolate our findings to all currently available lenses. Further investigation of the relationship between mechanical complications and fitting properties of daily disposables may be advantageous. More recent investigations with this modality would suggest a low risk of other complications. [17] Lens wear activities such as increased use of smart phones and disposable lens wear demographics, including the addition of multifocal and colored lens wearers, may also have changed during this time which may have impacted the risk profile in more recent years.

For other lens modalities, male gender [2,18,19] and younger age [3,20] are independent risk factors for microbial keratitis. The previously reported higher risk in males has previously been attributed to poorer compliance with lens wear and care and with greater risk taking behaviors, although it is possible that gender differences in ocular defence mechanisms contribute to this finding. Similarly, youth is associated with non-compliant lens wear and care behaviors. In the present study, considering the unweighted sample, wearers aged between 25–50 years had the highest risk but in the weighted sample this finding was no longer significant. It is conceivable that the reduced care requirements associated with daily disposable lenses may mitigate the impact of such risk factors.

Perhaps unexpectedly, reuse of daily disposable lenses was not a significant risk factor in univariate analysis, with 10% of both cases and controls reporting reuse of lenses. Given the low penetrance of lens re-use, it is not possible in this study to control for other potentially relevant factors such as overnight use and/or use of a storage case to further explore the impact of this behavior.

As with other lens modalities, [2, 21–23] smoking was a significant risk factor for any infection. Internet purchase was not associated with an increase in risk in daily disposable wearers, in contrast to a previous study of all lens modalities, [2] with 10% of both cases and control wearers obtaining their lenses via this supply route. Similarly 17% of cases and 15% of controls undertook a contact lens aftercare visit with their practitioner more than 12 months prior to completing the survey. Despite this finding, compliance with care recommendations in re-usable lens wearers can be improved at least temporarily, with advice and re-education. [24]

In this analysis, *Pseudomonas* spp were the most prevalent single species recovered in the corneal scrape analysis, which is consistent with previous studies. [25,26] However, the rate of recovery of all Gram negative bacteria, and particularly environmental organisms (those encountered in the human environment which are unlikely to be part of the normal human microbiota), including amoeba and fungi, was significantly lower in daily disposable wearers, compared with commensal organisms. This observation is consistent with the finding that more severe disease is associated with environmental pathogens [4,7] and disease severity is reduced in daily disposable wearers compared with other modalities. [2] It would be of interest to understand whether the microbial spectrum differs between occasional overnight use or strict daily use in daily disposable wearers but this analysis is limited by the small sample size.

Poor contact lens storage case hygiene practice, specifically failing to clean and air dry cases, is well-established as an independent risk factor for contact lens related microbial keratitis, [18,21,27–31] and is associated with a 4-fold increase in risk of the disease. [2] Failure to clean and air dry cases and not replacing cases frequently was associated with over 60% of the disease load in severe microbial keratitis in daily wear lens users. [32] It would be expected that the risk of microbial keratitis in daily disposable lens wear would be reduced compared with frequent replacement soft daily lens wear, but this appears to only be the case for more severe disease. [2] Considering the profile of organisms associated with daily disposable disease, corneal infections may be associated with organisms derived from lens handling. Bacterial adhesion and proliferation may occur on the relatively static environment of the back surface of the lens during wear. [33] Bacterial biofilm formation under these conditions may be of importance in prolonging the retention time of organisms at the ocular surface.

Combining data from similar but not identical studies has limitations. As wear populations and lens prescribing habits vary, some questions were tailored to each site, for example those describing socioeconomic class, thus combining results may not be truly representative of the total population. To provide a generalizable sample of cases and controls across the regions, a weighted sampling approach was adopted for statistical analysis. Though this method may lead to some factors being eliminated from the multivariate analysis, this was not the case. The significant factors of the weighted and unweighted method did not differ greatly. This approach is likely to best represent the populations and to improve generalizability of the results.

In conclusion, daily disposable contact lens use is associated with a low risk to the individual of any and particularly severe, microbial keratitis, with disease occurring at a rate of 1–2 per 10,000 wearers per year. [2] Increased exposure in daily wear, smoking and poor hand hygiene are significant independent risk factors for microbial keratitis with daily disposable contact lenses, which is consistent with previous findings in other wear modalities. In contrast, certain types of daily disposable lenses are associated with a lower risk. Daily disposable lens type, material properties and design are likely to play a role in the etiology of microbial keratitis in this wear modality. Environmental organisms are less likely to be associated with infection with this lens wear modality, which is consistent with a less severe disease phenotype and with the absence of a contact lens storage case.

## Supporting information

S1 File 2008 Dart et al Ophthalmology. (PDF)

S2 File 2008 Stapleton et al Ophthalmology. (PDF)

S3 File Univariate analysis. (XLSX)
