# Supplementary material for: Risk factors and causative organisms in microbial keratitis in daily disposable contact lens wear
Source: PLoS One. 2017 Aug 16;12(8):e0181343. doi: 10.1371/journal.pone.0181343 (PMC5558933; doi:10.1371/journal.pone.0181343)
Supplement: S1 File — (PDF) [file pone.0181343.s001.pdf]

Risk Factors for Microbial Keratitis with Contemporary Contact Lenses

A Case-Control Study

J. K. G. Dart, DM, FRCOphth,[1,2] *C. F. Radford, PhD,*[1] *D. Minassian, FRCOphth, MSc(Epidem),*[2]
S. Verma, MD, FRCOphth,[1] *F. Stapleton, PhD*[3]

Objective: To assess the relative risks (RR) of microbial keratitis (MK) for contemporary contact lens (CL) types and wearing schedules.

Design: A 2-year prospective case-control study begun in December 2003.

Participants: Cases were 367 CL wearers attending Moorfields Eye Hospital with proven or presumed MK. Controls were 1069 hospital controls, who were CL wearers with a disorder unrelated to CL wear, and 639 population-based controls who were CL wearers randomly selected from the Moorfields catchment area. Hospital patients completed a self-administered questionnaire; population-based controls were interviewed by telephone.

Testing: Multivariate analysis was done both for all cases of MK, and for the moderate and severe MK subgroups alone.

Main Outcome Measures: The RR for developing MK, and vision loss, for all lens types compared with planned replacement soft lenses (the referent).

Results: Compared with planned replacement soft lenses (the referent), the RR of MK was significantly increased with daily disposable (DD) CLs (RR, 1.56× [95% confidence interval (CI), 1.1–2.1]; $P = 0.009$) and differed between different brands of DD lens, was reduced for rigid lenses (RR, 0.16× [95% CI, 0.06–0.4]; $P<0.001$), and no different for silicone hydrogel or other types of soft lens. Although the risk of MK was higher overall among DD lens users, the risk of vision loss was less than for planned replacement soft CL users ($P = 0.05$); no DD lens users lost vision to the level of ≥20/40. The RR for overnight wear, for any lens type, was 5.4 times higher (95% CI, 3.3–10.9; $P<0.001$). Comparison of the DD soft CL types with planned replacement soft lenses (the referent), showed significant differences between brands for the risk of MK.

Conclusions: The risk of MK has not been reduced in users of DD and silicone hydrogel CLs. However, vision loss is less likely to occur in DD than in reusable soft CL users. Different brands of CL may be associated with significantly different risks of keratitis; understanding these differences should lead to the development of safer soft lenses. These findings suggest that lens/ocular surface interactions may be more important in the development of corneal infection than oxygen levels and CL case contamination.

Financial Disclosure(s): The authors have no proprietary or commercial interest in any materials discussed in this article. *Ophthalmology 2008;115:1647–1654 © 2008 by the American Academy of Ophthalmology.*

Microbial keratitis (MK) is a rare complication of contact lens (CL) wear, but is of interest because it is both a major cause of new cases of MK in the population, and the only sight-threatening complication of an otherwise safe method of vision correction.[1,2] Well-designed epidemiologic studies[1,3,4] have previously shown a 3-fold increase in the risk of MK for daily wear (DW) soft CL when compared with DW rigid CL and a 5-fold increase in the risk of MK when soft CL were worn overnight (sometimes termed extended or continuous wear). Both these differences in risk, and the annualized incidence, remained unchanged between 1989 and 1999.[3,4] Since these studies were undertaken, 2 new lens modalities have been introduced—daily disposable (DD) soft lenses in 1995, and in 1999 silicone hydrogel lenses, which are highly oxygen permeable and designed in some cases for continuous wear of up to 30 nights. Both lens types were introduced with the expectation that MK rates would be reduced. For DD lenses, this was hypothesized to be a result of the elimination of the CL hygiene and storage steps that have been shown to be a principal cause of microbial contamination in the CL user's environment,[5,6] and for silicone hydrogel lenses as a result of the reduction in the corneal hypoxia that was hypothesized to be a major risk factor for corneal infection.[7] Although clinical trials have reported an overall reduction in some adverse signs and symptoms for DD[8–10] and silicone hydrogel[11–13] lenses these have not had sufficient power to show a difference in risk for a rare complication such as MK. Meanwhile there

© 2008 by the American Academy of Ophthalmology
Published by Elsevier Inc.
ISSN 0161-6420/08/$–see front matter
doi:10.1016/j.ophtha.2008.05.003

Table 1. Definitions of Contemporary Contact Lens Types with Risks and Incidence of Microbial Keratitis Established in the Most Recent Well-Designed Studies

Contact Lens (CL) Type	Abbreviation	Description	Incidence	Relative Risk	Loss of Vision
Rigid	Rigid CL	Manufactured of rigid, gas-permeable polymers or polymethylmethacrylate	1.1:10 000 (CI, 0.6–1.7)*	RR, 1.0 (referent for other lens types)[4]	0.14:10 000
Soft	Soft CL	Manufactured from soft polymers	See below for 1–4 week replacement CL		
Silicone hydrogel	Not abbreviated	Highly oxygen-permeable soft polymer currently marketed for daily or ON wear and monthly disposal	18:10 000 (CI, 8.5–33.1) for ON[27]† DW not assessed		3.6:10 000
1–4 week planned replacement‡	Not abbreviated	Soft hydrogel lenses marketed for disposal after 1–4 weeks of wear	3.5:10 000 (CI, 2.7–4.5)[4] for Soft/Planned replacement CL daily wear	DW: RR 3.3 (CI, 1.9–6.1)	DW: 0.46:10 000
			20:10 000 (CI,10.3–35)[4] for Planned replacement CL ON wear	ON: RR 18.9 (CI, 10.3–35)	ON: 2.6:10 000
Daily disposable	Not abbreviated	Soft hydrogel lenses marketed for disposal after 1 day of wear	Not assessed		

CI = confidence interval; DW = daily wear; ON = overnight wear.
*Of the cases in this study,[4] 13% developed significant visual loss, similar to the figure of 14% in another recent study,[28] giving an approximate annualized risk of vision loss in the order of 5:100 000 for the use of DW soft lenses and 28:100 000 for ON soft lenses.
†Alternative term to continuous wear and extended wear.
‡Disposed of and replaced with a new lens on a planned schedule of between 1 and 4 weeks. These are sometimes known as frequent replacement CLs.

have been case reports of MK with both DD[14–19] and silicone hydrogel[20–26] lenses; a postmarket surveillance study found an annualized incidence rate of 18:10 000 (95% confidence interval [CI], 8.5–33.1) of MK among silicone hydrogel overnight wear users, similar to the rate for soft CL overnight wear in previous studies.[27]

Table 1 describes contemporary CL types, currently used in the global CL market, together with the best available estimates for the incidence, relative risk, and loss of vision associated with MK for each lens type, and wear schedule, for which data are available. There have been no well-designed epidemiologic studies to investigate the incidence or relative risks for MK with the new lens modalities. This case-control study was conducted at Moorfields Eye Hospital to measure the differences in risk for MK between currently available CL types and to identify any other associated risk factors. This study was designed to complement a national incidence study, also reported in this issue.[28] Both studies share the same definition of MK, severity classification, definitions of CL wearing schedules, and CL hygiene scoring system so that their results can be compared.

Methods

This was a prospective case-control study with data collection during the 2-year period beginning December 3, 2003.

Hospital Cases and Controls

Hospital patients completed a self-administered questionnaire providing data on possible risk factors, including demographic data, lens type, wear schedule, lens care, frequency of disposal, lens wear experience, frequency of practitioner aftercare, showering/face washing with lenses in, swimming, and smoking. A diagnosis for each questionnaire respondent was derived from the hospital notes. For patients with suspected MK, these notes were supplemented by a clinical data sheet completed by the Accident and Emergency (A&E) doctors, on which the lesion was drawn to scale, and any pain or anterior chamber activity were noted. Cases were CL wearers diagnosed with MK (as defined below). Hospital controls were CL wearers attending A&E as new patients with a disorder unrelated to CL wear.

Population-Based Controls

A telephone survey of the catchment population was undertaken every 6 months during the 2-year study period. Before each of the 4 surveys, the home postal districts of CL wearers who had attended Moorfields A&E during the previous 3 months were analyzed. For each postal district, the sampling frame was a complete list of household telephone numbers was provided by an address management software company (Hopewiser Ltd., Altrincham, WA15 8DG). From this frame, households were selected using simple random sampling, giving each household within the postal district equal probability of selection. For each postal district, the number of households selected was in proportion to the frequency with which this postal district occurred among the list of A&E patients. All CL users within each selected household were eligible for interview. Sampling continued until the required number of controls was obtained. Sample size calculations, using data from a 3-month pilot study completed immediately before this study was started, required identification of 160 CL wearers in the catchment population in each of the 4 surveys. Trained telephone interviewers called numbers from the household telephone numbers list on weekday evenings (excluding Fridays) and Saturday mornings. Up to 4 attempts to obtain complete data from each

household were allowed. A scripted version of the Moorfields Eye Hospital A&E questionnaire was carried out with any CL users in the household. Eligible individuals completing the questionnaire were used as nonhospital controls.

Exclusions

Cases or controls were excluded if they had insufficient questionnaire data despite attempts to contact them to clarify and/or complete data, had not used a CL during the previous 30 days, had a medical indication for CL wear, or had any previous attendance at Moorfields.

Study Definitions

Table 2 (available online at http://aaojournal.org) summarizes the study definitions common to both this study and to the companion study on the incidence of CL related MK in Australia.[28] These definitions are described below.

Microbial Keratitis

Microbial keratitis was defined by either a positive corneal culture or a corneal infiltrate and overlying epithelial defect with ≥1 of the following features: (i) any part of the lesion being within the central 4 mm of the cornea, (ii) uveitis, or (iii) pain. Cases were classified as "severe" if they resulted in significant visual loss (final visual acuity, with spectacles or pinhole, of ≤20/40) without any other attributable cause. Cases without significant visual loss were defined as "moderate" if they had one or more of the following features: (i) a positive corneal culture, (ii) a lesion within or overlapping the central 4 mm of the cornea, (iii) hypopyon, or (iv) ≥4 hospital visits. All other cases were classified as "mild." Other corneal infiltrates, with or without overlying epithelial defects, that did not meet these criteria for MK were classified as corneal infiltrative events (corneal ulcers or infiltrates occurring in the absence of infection).[29]

Wear Schedules

Overnight wear occurs with all of these lens types, either in a regular pattern (at least once per week) or on an occasional (and often unplanned) basis. For this reason, all CL types were subdivided into 3 wear schedule categories: DW (no overnight use admitted), occasional overnight wear (overnight use less often than once per week), and overnight wear (habitual overnight use of once per week or more). Overnight use included those users who were wearing lenses for continuous periods of up to 30 days (sometimes termed extended or continuous wear).

Contact Lens Hygiene Compliance

Lens hygiene compliance is an important variable and our evaluation used 12 multiple choice questions. A composite hygiene score for each subject was allocated using a weighted scoring system for each of the 4 key areas of CL hygiene: disinfection or lens disposal (0–20), storage case replacement (0–4), storage case hygiene (0–8), and hand washing before handling CLs (0–8). The maximum score was 40, for a DD lens user who disposed of their lens on removal (20 points), who never stored it (12 points), and who always washed and dried their hands before lens handling (8 points). The minimum score (0 points) was obtained by those reusing lenses without disinfection solutions (0 points), who never emptied and air dried their case (0 points), who replaced the case less than annually (0 points) and who only washed their hands "sometimes" (0 points). Daily disposable lens users lost 32 points (all points except the hand hygiene score) if they reused their lenses at all (some of these lens types are specifically designed for 1-day wear with characteristics that change with time).

Statistical Analysis

The outcome of primary interest was MK, as defined. A secondary outcome in the case-control analysis was moderate to severe MK combined (excluding the mild cases from analysis). This was done to establish whether the inclusion of mild cases, which could have included a proportion of corneal infiltrative events, might have a substantial effect on the findings. Among the MK cases, severe MK (with vision loss of ≥20/40) was also considered as an outcome, and its frequency compared between lens types. The candidate pool of possible risk factors considered in the building of regression models comprised demographic factors, lens type, wear schedule, frequency of wear, lens care, frequency of disposal (hygiene score), lens wear experience, frequency of practitioner aftercare, showering/face washing with lenses in, swimming, and smoking. The planned replacement soft lens was used as the referent in several comparisons because of the clinical interest in differences between soft lens types. Where appropriate, chi-square tests for comparing proportions, and *t* tests for comparison of means, were used. Binomial logistic regression (for binary outcomes) was used to estimate odds ratios as measures of association. Odds ratios are reported as estimates of relative risk throughout this study. Analyses where odds ratio estimates were adjusted for effect of other covariates (potential confounders) are referred to throughout this paper as multivariate analysis. The main analysis included all MK cases. Interactions between CL type and covariates were assessed through classical methods (testing homogeneity of odds ratios across the covariate strata), and through likelihood ratio tests comparing appropriate logistic models with and without the interaction term. These were limited to interactions thought to be biologically feasible. The regression diagnostics included the Hosmer-Lemeshow goodness-of-fit test, and calculation of the area under the receiver operating characteristic curve. The statistical software used was STATA (Stata Corporation, TX). The study was approved by the Local Ethics Committee.

Results

The study design resulted in 367 cases and 2075 controls from 185 of 220 (84%) of Greater London postcodes (population of Greater London[30] 7 172 000) as well as from 241 UK postal districts outside London.

Table 3 shows the demographic data and the distribution of lens types and wear schedules for the 367 MK cases and the 2 different control groups included in the study. Another 241 CL users had corneal infiltrative events[29] and were not included in this study. There were 77 hospital cases and controls who were excluded owing to missing or ambiguous data regarding lens type, wear schedule, and/or hygiene.

Table 4 (available online at http://aaojournal.org) shows the severity of MK among the cases and the organisms isolated. Of the 349 cases, 15 (4%) were categorized as severe. An additional 18 of 367 (4.9%) had an acuity of ≤20/40 at their last visit but were unclassified because of loss to follow-up or discharge before resolution (n = 13), a history of amblyopia (n = 2) or retinal problems (n = 2), or having unrecorded visual acuity at their final visit (n = 1). There were 179 (51%) moderate and 155 (44%) mild cases of MK. Cultures were carried out in 123 cases; the remainder had lesions considered too small to justify culture and were treated with intensive broad-spectrum antibiotic drops. Daily disposable

Table 3. Demographic Data, Contact Lens (CL) Types, and Numbers Using Daily (DW) or Overnight (ON) Wear Schedules for Cases and Control Groups

	Case and Control Groups (n)			
	Microbial Keratitis (n = 367)	*Hospital Controls (n = 1069)*	*Nonhospital Controls (n = 639)*	*Total (n = 2075)*
Demographic data				
Mean age (range)	32.3 (15–71)	34.8* (14–78)	36.7* (13–77)†	
Male (%)	173 (47)	363 (34)	223 (36)‡	
Occupation SOC; Major groups:1–3:4–9 (%)	250:85 (75:25)§	728:261 (74:26)¶	391:162 (71:29)#	
Contact lens types and wear schedule				
Daily disposable (n = 813)				
DW	70 (55)	311 (75)	191 (85)	567 (70)
Occasional ON	65 (41)	98 (25)	62 (23)	225 (28)
ON	10 (6)	4 (1)	7 (3)	21 (2)
Planned replacement (n = 766)				
DW	85 (53)	284 (74)	198 (74)	572 (75)
Occasional ON	50 (39)	95 (23)	29 (13)	174 (23)
ON	7 (6)	9 (2)	4 (92)	20 (2)
Silicone hydrogel (n = 188)				
DW	8 (12)	43 (48)	17 (50)	68 (36)
Occasional ON	7 (11)	19 (21)	4 (12)	30 (16)
ON	49 (77)	38 (31)	13 (38)	90 (48)
Other soft CL (n = 95)				
DW	8 (73)	46 (82)	24 (86)	78 (82)
Occasional ON	3 (27)	10 (18)	4 (14)	17 (18)
ON	0	0	0	0
Rigid (n = 213)				
DW	5 (100)	113 (93)	81 (94)	199 (93.5)
Occasional ON	0	8 (6)	5 (6)	13 (6)
ON	0	1 (1)	0	1 (0.5)

SOC = The Standard Occupational Classification 2000, Office for National Statistics. Groups 1–3 are Managers and Senior Officials, Professional Occupations, and Associate Professional and Technical Occupations.
Occasional ON wear is defined as less often than 1 time per week, whereas ON wear is defined as ≥1 night per week.
*Significantly different (*t* test); $P = 0.0028$.
†Four unknown.
‡Seventeen unknown.
§Thirty-two unknown.
¶Eighty unknown.
#Eighty-six unknown.

lens users experienced less severe keratitis than those using planned replacement soft CLs, both when used for DW only ($P = 0.0077$) and when used for both daily and overnight wear ($P = 0.0393$; Table 4).

Multivariate Analysis

In the multivariate analysis, there was minor variation in odds ratio estimates (reported as estimates of relative risk) depending on whether the hospital or nonhospital control group was used, but the main findings were very similar and the final analysis reported here uses all controls combined. In addition, the analysis was done both for all MK cases and separately for the combined subgroups of severe and moderate keratitis (excluding the 155 mild cases). This was done to establish whether the inclusion of mild cases, which could have included a proportion of corneal infiltrative events, might have a substantial effect on the findings. Few differences were found and the results reported in Tables 5 and 6 are for all cases of MK, with footnotes summarizing the differences in the findings for the severe and moderate MK subgroup, where these differ.

Table 5 shows all the major associations with MK. After adjustment for the effect of the other factors in the table, DD soft lenses carried a higher risk of MK compared to planned replacement soft lenses of 1.5 times, and rigid lenses a 5-fold reduced risk. In the severe and moderate MK subgroup analysis, this difference between DD and planned replacement soft lenses was not statistically significant. Overnight wear, of any lens type, increased the risk of MK by 5-fold. The increased risks of MK associated with the use of hypermetropic lens corrections, younger users, and males were of borderline significance, or not significantly different, in the subgroup analysis of severe and moderate MK.

Table 6 shows the results of the analysis for DW lens users only and includes the analysis of risks for MK associated with different brands of DD lens. Compared with planned replacement soft lenses the risk of developing MK was significantly higher: 3.24× with Dailies (CIBA Vision, Duluth, GA), 2.48× with Soflens One Day (Bausch & Lomb, Rochester, NY), and 2.10× for a group of other/unidentified brands of DD lenses. In the subgroup analysis of severe and moderate MK, these differences were unchanged for Dailies (CIBA Vision) but of borderline statistical significance for Soflens One Day (Bausch & Lomb) and other brands. Over half of the other brands group (104/154) was composed of Boots Dailies and Easyvision 1 Day (Specsavers); these companies rebrand DD lenses from CIBA Vision and Bausch & Lomb, but neither the

Table 5. Independent Risk Factors for Major Potential Associations with Microbial Keratitis (MK) in Contact Lens (CL) Users, Derived from Multiple Logistic Regression Analysis

Risk Factor	Relative Risk (RR)	95% CI	P Value
CL type			
Planned replacement soft	1.00 (referent)		
Silicone hydrogel	1.16	0.73–1.86	0.525
Other soft CL	0.87	0.44–1.73	0.698
Daily disposable	1.56	1.12–2.17	0.009*
Rigid	0.16	0.06–0.40	<0.001
Wear schedule			
DW only	1.00 (referent)		
Occasional ON	1.87	1.42–2.46	<0.001
ON	5.28	3.26–8.56	<0.001
Days of CL wear per week			
≤2	1.00 (referent)		
3–5	3.46	1.82–6.56	<0.001
6–7	6.05	3.34–10.96	<0.001
Reason for wearing CL			
Myopia	1.00 (referent)		
Hypermetropia	1.77	1.08–2.91	0.024†
To alter eye color	2.19	0.75–6.37	.0152
Hand washing before cleaning			
Always	1.00 (referent)		
Not always	1.49	1.13–1.97	0.005
Hygiene score			
Excellent (33–40)	1.00 (referent)		
Good (27–32)	0.83	0.59–1.18	0.300
Moderate (22–26)	1.00	0.61–1.64	0.992
Poor (0–21)	1.12	0.75–1.66	0.584
Age (y)			
≤49	1.00 (referent)		
≥50	0.45	0.27–0.74	0.002‡
Gender			
Female	1.00 (referent)		
Male	1.48	1.15–1.89	0.002§

CI = confidence interval; DW = daily wear; ON = overnight wear.
The RR for each factor is adjusted for the effects of the other factors.
A subgroup analysis was also done for severe and moderate MK alone (excluding the 155 cases defined as mild MK).
Differences in the results for the subanalysis of severe and moderate keratitis only:
*Not significantly different: RR, 1.37 (0.89–2.09); $P = 0.148$.
†Borderline: RR, 1.8 (0.98–3.30); $P = 0.059$.
‡Not significantly different: RR, 0.57 (0.31–1.07); $P = 0.079$.
§Not significantly different: RR, 1.3 (0.94–1.79); $P = 0.105$.

companies nor the manufacturers were able to identify these lenses by manufacturer's brand. In this other brands group, it is not possible to ascribe the increased risk to any particular brand or brands of lens.

We also analyzed differences between brands of planned replacement soft lenses. There were 13 different brands together with a mixture of other brands of lenses. There were no differences between brands when these lenses were used for DW only. However, when all the planned replacement lens users were included in the analysis, including those using the lenses for occasional overnight and overnight wear, 1 lens type, Soflens 66 (Bausch & Lomb), was significantly less associated with MK than 4 of the other brands (Acuvue 2 [Johnson & Johnson, Langhorne, PA]: RR, 3.47; 95% CI, 1.11–12.99; $P = 0.034$; Biomedics 55 UV [Ocular Sciences, Concord, CA]: RR, 3.47; 95% CI, 1.20–11.76; $P = 0.046$; Enhance [Vision Express, Lenton, Nottingham, UK]: RR, 4.37; 95% CI, 1.07–17.89; $P = 0.040$; and Surevue [Johnson & Johnson]: RR, 3.85; 95% CI, 1.01–14.67; $P = 0.048$). These RRs are adjusted for effect of other covariates (age, gender, overnight wear, and days per week wearing CL, reason for CL, hand washing, and hygiene score). No differences were found between the brands of silicone hydrogel lenses for MK.

Last, we compared differences in risk for MK between users of planned replacement soft lenses and silicone hydrogel lenses for habitual overnight wear and adjusted the multivariable analysis for the maximum number of consecutive nights of CL wear. This was done because the mean period of overnight wear was longer in the 90 silicone hydrogel lens users (mean, 32.4; median, 28 for silicone hydrogel users) compared with 20 planned replacement soft CL users (mean, 28.4; median, 2.5) developing MK. No differences were shown between the lens types, although this analysis had limited power to show differences.

Results of the regression diagnostics for the final completed models were as follows: The Hosmer-Lemeshow goodness-of-fit test P values ranged from 0.840 to 0.915, indicating that models fit quite well. The areas under the receiver operating characteristic curves ranged from 72% to 76%, indicating adequate discriminatory performance of the models (≥80% considered as excellent discrimination).

Table 6. Comparisons of Risks of all Microbial Keratitis for Daily Wear Only, Adjusted for Effects of Other Factors*

Risk Factor	Relative Risk (RR)	95% CI	*P*
CL type/brand			
Planned replacement soft	1.00 (referent)		
Silicone hydrogel	0.92	0.41–2.04	0.832
Other soft CL	0.99	0.44–2.20	0.979
Daily disposable CL brands			
1-Day Acuvue (Johnson & Johnson)	0.68	0.35–1.34	0.266
Dailies (CIBA Vision)	3.24	1.86–5.64	<0.001
Soflens One Day (Bausch & Lomb)	2.48	1.03–5.96	0.043†
Other/Unidentified	2.10	1.19–3.72	0.011‡
Rigid	0.20	0.08–0.52	0.001

*Other factors include days of CL wear per week, reason for wearing CL, hand washing before CL handling, hygiene score, age, and gender.
A subgroup analysis was also done for severe and moderate MK alone (excluding the 79 cases defined as mild MK). Differences in the results for the subanalysis of severe and moderate keratitis only:
†Borderline difference: RR, 2.73 (0.94–7.95); $P = 0.066$.
‡Borderline difference: RR, 2.09 (1.00–4.37); $P = 0.500$.

Discussion

This study measured the risks of MK, for the first time, for the DD soft CLs and silicone hydrogel lenses that have been introduced in the 10 years since the completion of the previously published epidemiologic studies on MK in CL wearers.[1,3,4] The design of this study has provided the largest MK case series to date and a sample of patients from a large catchment area, which allows extrapolation of our findings to CL users in the United Kingdom and other similar environments. A limitation of the study design is that the size of the catchment population for this study cannot be assessed with precision, because of loss of both cases and controls to the many surrounding hospitals, so that we are unable to measure the incidence of MK in CL users; instead, this has been done in the complementary Australian national incidence study.[28] The planned replacement soft lens, rather than the gas-permeable rigid lens, was chosen as the referent for several comparisons because there is more clinical interest in the differences between soft lens types; soft lenses have been used as referent in several other studies.[3,28,31]

This study has shown no change in the size of the relative risks measured by the previous epidemiologic studies, for the lens types that were available during those periods. The principal new findings relate to the newly introduced lens types for which there is no substantive epidemiologic data for the risks of MK.

Severe MK, resulting in vision loss ≥20/40, is fortunately a small subset of MK in lens users. Among DD lens users, none lost vision at this level. There was a statistically significant reduction in the severity of MK in DD lens users compared with that in planned replacement and other soft lens users. However, with this exception, DD lens wear has not resulted in the anticipated reduction in MK among DW soft lens users and the risk of developing any category of MK carried a significantly higher risk for DD lens use, compared with the use of planned replacement soft lenses. This increased risk of MK was also present for some brands of DD soft lens in a subgroup analysis restricted to severe and moderate MK. In the severe and moderate MK subgroup analysis, this difference was not significant when all brands of DD lens were pooled, probably both because 1 brand with a reduced risk (Acuvue 1-Day) balanced the increased risks shown with other brands and also because of the reduced power of the subgroup analysis to show a difference. Differences between brands of planned replacement soft lens were also present, but less so, and were not significant when lenses were used for strict DW only.

Possible reasons for our findings include differences in fit and material between brands of DD lens, the tendency to prescribe DDs for some patients who have had difficulties tolerating, or carrying out hygiene procedures for, reusable lenses and the use of DDs for activities that may predispose to infection, such as swimming and other sports resulting in eye contamination with soil and water. Previous studies have reported patients finding DD lenses more difficult to handle than planned replacement lenses,[9] and the more rigid Dailies (CIBA Vision) lenses harder to remove than the Acuvue 1-Day (Johnson & Johnson) lens.[31] Difficulty with lens handling can lead to corneal abrasions and, in the case of "stuck" lenses, unavoidable overnight wear (controlled for in this study), both of which predispose to corneal infection. This study has also shown that 30% of DD lens users are using these for occasional or regular overnight wear, an off-indication use that increases their risk of developing MK. The significantly milder keratitis in DD lens users, compared with that in planned replacement soft CL users, is probably due to the presence of pathogenic organisms in the CL cases of planned replacement soft CL users[5,6] that are less often found elsewhere in the CL user's environment. Some of the brands have altered their lens parameters since the study was conducted and the findings reported here, for individual brands, may not be representative of lenses currently in use.

Silicone hydrogel lenses were found to have no significant effect on the risk or severity of MK. This conflicts with a recent study reporting that, compared with soft CL over-

night users, overnight silicone hydrogel lens users have a 5 times lower risk of severe MK[32] and that corneal infiltrative events are significantly less severe.[33] These studies utilized indirect estimates of the size of the study population, which we believe invalidates their conclusions.[34] A conclusion of the recent postmarket surveillance study[27] was that, although the rate of MK was similar to that reported by earlier studies for soft CL overnight use, the silicone hydrogel lenses were used for much longer periods, suggesting that the high oxygen permeability of silicone hydrogel lenses may be reducing the risk of MK with overnight wear; we have not been able to demonstrate this in the small subgroup analysis of habitual ON lens users. However, this subgroup analysis has limited power and there may be a benefit associated with the use of silicone hydrogel lenses for longer periods of wear that we cannot demonstrate.

Our results have implications for our understanding of the pathogenesis of keratitis in CL users. The disappointing finding that silicone hydrogel CL use has no major effect on MK suggests that other, possibly less easily modifiable factors, such as tear film stagnation, ocular surface compartmentalization behind the lens, and reduced corneal epithelial cell turnover with soft lens wear[35] may play a more significant role in the development of corneal infection than corneal hypoxia. These factors may also be more critical than the effect of exposure to a contaminated CL case, as shown by the finding that no brand of DD CL has had the expected effect of reducing the risk of MK. On the other hand, the finding that the introduction of the DD CL has had a brand-dependent effect on the risk of MK suggests that a difference in soft CL design and/or polymer, rather than its method of use, can modify susceptibility to MK. This has further implications, both for our understanding of the pathogenesis of MK in CL users and/or future lens design.

Patients wishing to correct their vision with an alternative to spectacles have available either CL wear or laser refractive surgery, of which the most widely used procedure is probably LASIK. Vision loss with LASIK has never been investigated from the population perspective in the same way as CL wear and the data that are available come from small, often single surgeon, case series. Current data shows that CLs can be worn safely with a substantially lower risk of vision loss than refractive surgery procedures and without the additional problems of glare, dry eye, and poor quality of night vision.[27]

One of the strengths of this study is the similarities in the findings between this and the concurrent companion Australian incidence study,[28] also published in this issue. These similarities and the differences have been summarized at the end of the discussion in that paper.

To maximize safe CL wear, practitioners should ensure that their patients wear their lenses according to the recommended wearing schedule; are properly instructed in lens handling, lens case hygiene, and replacement; and are aware of the importance of good hand hygiene, the increased risk associated with the overnight use, or more frequent daily use, of soft lenses.

Acknowledgments. The authors thank the following steering committee members—O. Schein, D. Cavanagh, B. Holden, H. Taylor, D. Fonn, and J. McNally—for protocol and data review, and Dr Eric Beck, Ms Valerie Saw, and Ms Alison Mathews for reviewing the manuscript.

The authors were responsible for the design and conduct of the study; had access to and carried out the collection, management, analysis, and interpretation of all the data; and then undertook the preparation, review, and approval of the manuscript. A draft of the manuscript was provided to CIBA Vision Corporation and the Institute for Eye Research, before publication. Modifications were limited to clarification and grammatical changes.

References

1. Dart JK, Stapleton F, Minassian D. Contact lenses and other risk factors in microbial keratitis. Lancet 1991;338:650–3.
2. Keay L, Edwards K, Naduvilath T, et al. Microbial keratitis: predisposing factors and morbidity. Ophthalmology 2006;113: 109–16.
3. Schein OD, Glynn RJ, Poggio EC, et al, Microbial Keratitis Study Group. The relative risk of ulcerative keratitis among users of daily-wear and extended-wear soft contact lenses: a case-control study. N Engl J Med 1989;321:773–8.
4. Cheng KH, Leung SL, Hoekman HW, et al. Incidence of contact-lens-associated microbial keratitis and its related morbidity. Lancet 1999;354:181–5.
5. Mayo MS, Cook WL, Schlitzer RL, et al. Antibiograms, serotypes, and plasmid profiles of *Pseudomonas aeruginosa* associated with corneal ulcers and contact lens wear. J Clin Microbiol 1986;24:372–6.
6. Stapleton F, Dart JK, Seal DV, Matheson M. Epidemiology of *Pseudomonas aeruginosa* keratitis in contact lens wearers. Epidemiol Infect 1995;114:395–402.
7. Dart J. Extended-wear contact lenses, microbial keratitis, and public health. Lancet 1999;354:174–5.
8. Hamano H, Watanabe K, Hamano T, et al. A study of the complications induced by conventional and disposable contact lenses. CLAO J 1994;20:103–8.
9. Solomon OD, Freeman MI, Boshnik EL, et al. A 3-year prospective study of the clinical performance of daily disposable contact lenses compared with frequent replacement and conventional daily wear contact lenses. CLAO J 1996;22: 250–7.
10. Sankaridurg PR, Sweeney DF, Holden BA, et al. Comparison of adverse events with daily disposable hydrogels and spectacle wear: results from a 12-month prospective clinical trial. Ophthalmology 2003;110:2327–34.
11. Brennan NA, Coles ML, Comstock TL, Levy B. A 1-year prospective clinical trial of balafilcon A (PureVision) silicone-hydrogel contact lenses used on a 30-day continuous wear schedule. Ophthalmology 2002;109:1172–7.
12. Chalmers RL, Dillehay S, Long B, et al. Impact of previous extended and daily wear schedules on signs and symptoms with high Dk lotrafilcon A lenses. Optom Vis Sci 2005; 82:549–54.
13. Riley C, Young G, Chalmers R. Prevalence of ocular surface symptoms, signs, and uncomfortable hours of wear in contact lens wearers: the effect of refitting with daily-wear silicone hydrogel lenses (Senofilcon A). Eye Contact Lens 2006; 32:281–6.
14. Hingorani M, Christie C, Buckley RJ. Ulcerative keratitis in a person wearing daily disposable contact lenses [letter]. Arch Ophthalmol 1995;79:1138.
15. Woodruff SA, Dart JK. *Acanthamoeba* keratitis occurring with daily disposable contact lens wear [letter]. Br J Ophthalmol 1999;83:1088–9.

16. Blades KJ, Tomlinson A, Seal D. *Acanthamoeba* keratitis occurring with daily disposable contact lens wear [letter]. Br J Ophthalmol 2000;84:805.
17. Choi DM, Goldstein MH, Salierno A, Driebe WT. Fungal keratitis in a daily disposable soft contact lens wearer. CLAO J 2001;27:111–2.
18. Su DH, Chan T, Lim L. Infectious keratitis associated with daily disposable contact lenses. Eye Contact Lens 2003;29:185–6.
19. Munneke R, Lash SC, Prendiville C. A case of *Pseudomonas* corneal ulcer in an occasional use daily disposable contact lens wearer. Eye Contact Lens 2006;32:94–5.
20. Lim L, Loughnan MS, Sullivan LJ. Microbial keratitis associated with extended wear of silicone hydrogel soft contact lenses [letter]. Br J Ophthalmol 2002;86:355–7.
21. Skotnitsky C, Jalbert I, O'Hare N, et al. Case reports of three atypical infiltrative keratitis events with high DK soft contact lens wear. Cornea 2002;21:318–24.
22. Lee KY, Lim L. *Pseudomonas* keratitis associated with continuous wear silicone-hydrogel soft contact lenses: a case report. Eye Contact Lens 2003;29:255–7.
23. Holden BA, Sweeney DF, Sankaridurg PR, et al. Microbial keratitis and vision loss with contact lenses. Eye Contact Lens 2003;29(suppl):S131–4.
24. Schornack MM, Peterson D. *Staphylococcus aureus* ulcer associated with continuous wear of silicone hydrogel contact lenses. Eye Contact Lens 2006;32:72–4.
25. Whiting MA, Raynor MK, Morgan PB, et al. Continuous wear silicone hydrogel contact lenses and microbial keratitis. Eye 2004;18:935–7.
26. Syam P, Hussain B, Hutchinson C. Mixed infection (*Pseudomonas* and coagulase negative staphylococci) microbial keratitis associated with extended wear silicone hydrogel contact lens [letter]. Br J Ophthalmol 2004;88:579.
27. Schein OD, McNally JJ, Katz J, et al. The incidence of microbial keratitis among wearers of a 30-day silicone hydrogel extended-wear contact lens. Ophthalmology 2005;112:2172–9.
28. Stapleton F, Keay L, Edwards K, et al. The incidence of contact lens-related microbial keratitis in Australia. Ophthalmology 2008;115:1655–62.
29. Sweeney D, Jalbert I, Covey M, et al. Clinical characterization of corneal infiltrative events observed with soft contact lens wear. Cornea 2003;22:435–42.
30. National Statistics. Census 2001: London. Available at: http://www.statistics.gov.uk/census 2001/pop2001/london.asp. Accessed January 14, 2008.
31. Inaba M. 1-Day Acuvue vs. Focus Dailies: a comparison of comfort, user preference, and incidence of corneal complications. CLAO J 2000;26:141–5.
32. Morgan PB, Efron N, Hill EA, et al. Incidence of keratitis of varying severity among contact lens wearers. Br J Ophthalmol 2005;89:430–6.
33. Efron N, Morgan PB, Hill EA, et al. Incidence and morbidity of hospital-presenting corneal infiltrative events associated with contact lens wear. Clin Exp Optom 2005;88:232–9.
34. Stapleton F, Keay L, Edwards K, et al. Comment: Incidence of keratitis of varying severity amongst contact lens wearers [electronic letter]. BJO Online. Available at: http://bjo.bmj.com/cgi/eletters/89/4/430#725. Accessed April 3, 2008.
35. Fleiszig SM, Evans DJ. Contact lens infections: can they ever be eradicated? Eye Contact Lens 2003;29(suppl):S67–71.

Footnotes and Financial Disclosures

Originally received: October 28, 2007.
Final revision: April 27, 2008.
Accepted: May 1, 2008.
Available online: July 2, 2008. Manuscript no.: 2007-1401.

[1] Moorfields Eye Hospital NHS Foundation Trust, London, United Kingdom.

[2] Institute of Ophthalmology, University College London, London, United Kingdom.

[3] School of Optometry and Visual Science University of New South Wales & Institute of Eye Research, Sydney, Australia.

Financial Disclosure(s):
None of the authors have any personal conflicts of interest.

Supported by the Institute for Eye Research, University of New South Wales, Sydney, Australia from a project grant made by CIBA Vision Corporation.

Correspondence:
John K.G. Dart, DM, FRCOphth, Moorfields Eye Hospital, 162 City Road, London EC1V 2PD. E-mail: j.dart@ucl.ac.uk.

Table 2. Summary of Study Definitions for This Study and the Companion Incidence Study[28]

Term	Definition
Keratitis	
Microbial keratitis (MK)	A positive corneal culture or corneal infiltrate and overlying epithelial defect with ≥1 of the following: (i) any part of the lesion within or overlapping the central corneal 4 mm; (ii) uveitis; (iii) pain.
Severe MK	Vision loss of ≥20/40 (final VA with spectacles or pinhole) without any other attributable cause. In the Australian study, estimates of vision loss were also defined as loss of ≥2 lines of BCVA.
Moderate MK	No significant vision loss, with ≥1 of the following: (i) a positive corneal culture; (ii) lesion within or overlapping the central corneal 4 mm; (iii) hypopyon; (iv) ≥4 hospital visits.
Mild MK	All other cases of MK.
Corneal infiltrative events[33]	Any infiltrate and/or ulcer not meeting the MK criteria.
Wear schedules	
Daily	No overnight use ever admitted.
Occasional ON	ON use ≤1 time per week.
ON	Habitual ON lens users of once a week a week or more. This includes those users of 30-day CW CLs (sometimes known as extended wear CLs).
Hygiene scoring system	A composite hygiene score (range, 0–40) for each subject used a weighted scoring system for each of the 4 key areas of contact lens hygiene: (i) disinfection or lens disposal (0–20); (ii) storage case replacement (0–4); (iii) storage case hygiene (0–8); and (iv) hand washing before handling CLs (0–8).
Maximum score (40) example	A daily disposable CL user (i) disposing of the CL on removal (20 points); (ii and iii) never storing it (12 points); and (iv) always washing and drying hands before CL handling.
Minimum score (0) example	Reusing lenses without disinfection (0 points), no CL case emptying or drying (0 points), case replacement more than once a year (0 points), infrequent hand washing (0 points). *Note*. Daily disposable CL users lost 32 points (all points except the hand hygiene score) if they reused their CLs at all (some of these lens types are specifically designed for 1-day wear with characteristics that change with time).
Cases	
Inclusion criteria	Cases were CL users within 30 days of developing disease, and 15–64 years old, and using CLs for the correction of low refractive errors.
Exclusion criteria	Medical indications for CL wear.
Controls	See details in the Methods sections of both papers.

CL = contact lenses; DW = daily wear; ON = overnight wear.

Table 4. Distribution of Severity and Microbial Isolate for Keratitis (MK) between Contact

MK Severity (n, %)	CL Types and Use of DW					
	Daily Disposable		*Planned Replacement*		*Silicone Hydrogel*	
	DW	*ON*	*DW*	*ON*	*DW*	*ON*
Severe (15, 4)	0	3	6	3	0	2
Moderate (179, 51)	43	29	37	30	5	28
Mild (155, 44)	39	37	25	22	2	24
Unknown (18)	3	6	2	2	1	2
Organism	Distribution of microbial isolates between					
Bacterial	2 *Pseudomonas* sp<br>5 *Staphylococcus* sp.	6 *Pseudomonas* sp<br>3 *Staphylococcus* sp.<br>1 *S marcescens*<br>1 *S viridans*	7 *Pseudomonas* sp<br>5 *Staphylococcus* sp.<br>2 *S marcescens*<br>2 *Corynebacterium* sp	4 *Pseudomonas* sp<br>2 *Staphylococcus* sp.	2 *Pseudomonas* sp<br>1 *Staphylococcus* sp.	6 *Pseudomonas* sp<br>3 *Staphylococcus* sp.<br>1 *S viridans*<br>1 *Klebsiella* (bilateral)
Acanthomoeba	0	0	1	1	0	1
Fungi	1 *Fusarium dimerum*			I *Acremonium*		*1 T mucoides +* *Candida* sp

Occasional and habitual ON CL users have been combined for this table.
Daily disposable lenses were less associated with severe MK compared with planned replacement lenses for both DW and when DW and ON are combined: MK versus moderate MK for DW daily disposable versus DW planned replacement lenses users, $P = 0.0077$ (Fisher exact test; 2 tailed).

Lens (CL) Types and Daily (DW) or Overnight (ON) Schedules

or ON Wear for Each Type				
Other Soft CL		*Rigid CL*		
DW	*ON*	*DW*	*ON*	**Distribution of Isolates (n = 60) from 123 Cultures**
0	0	1	0	7 *Pseudomonas* sp<br>1 *Staphylococcus* sp<br>1 *Streptococcus viridans*<br>1 *Klebsiella*<br>1 *Acanthamoeba*
4	2	1	0	20 *Pseudomonas* sp<br>18 *Staphylococcus* sp<br>1 *Streptococcus* sp<br>3 *Serratia marcescens*<br>2 *Corynebacterium* sp<br>2 *Acanthamoeba*<br>1 *Acremonium* sp<br>1 *Fusarium dimerium*<br>1 *Trichosporon* + *Candida* sp
3	0	3	0	None isolated
1	1	0	0	None isolated
different types and schedules of CL wear (n = 60)				
0	0	0	0	

severe MK versus moderate/mild MK for all daily disposable versus planned replacement lenses users $P = 0.0393$ (Fisher exact test, 2 tailed); and severe
