# Supplementary material for: Risk factors and causative organisms in microbial keratitis in daily disposable contact lens wear
Source: PLoS One. 2017 Aug 16;12(8):e0181343. doi: 10.1371/journal.pone.0181343 (PMC5558933; doi:10.1371/journal.pone.0181343)
Supplement: S2 File — (PDF) [file pone.0181343.s002.pdf]

The Incidence of Contact Lens–Related Microbial Keratitis in Australia

Fiona Stapleton, PhD,[1,2,3] Lisa Keay, PhD,[2,3] Katie Edwards, BAppSc (Optom),[2,3] Thomas Naduvilath, PhD,[1,2,3] John K. G. Dart, DM,[4,5] Garry Brian, FRANZCO,[3,6] Brien A. Holden, DSc[1,2,3,6]

Objective: To establish the absolute risk of contact lens (CL)-related microbial keratitis, the incidence of vision loss and risk factors for disease.

Design: A prospective, 12-month, population-based surveillance study.

Participants: New cases of CL-related microbial keratitis presenting in Australia over a 12-month period were identified through surveillance of all ophthalmic practitioners (numerator). Case detection was augmented by records' audits at major ophthalmic centers. The denominator (number of wearers of different CL types in the community) was established using a national telephone survey of 35 914 individuals.

Testing: Cases and controls were interviewed by telephone to determine subject demographics and CL wear history. Visual outcomes were determined 6 months after the initial event. Annualized incidence and confidence intervals (CI) were estimated for different severities of disease and multivariable analysis was used in risk factor analysis.

Main Outcome Measures: Annualized incidence (with CI) of disease and vision loss by CL type and wear modality and identification of independent risk factors.

Results: We identified 285 eligible cases of CL-related microbial keratitis and 1798 controls. In daily wear rigid gas-permeable CL wearers, the annualized incidence per 10 000 wearers was 1.2 (CI, 1.1–1.5); in daily wear soft CL wearers 1.9 (CI, 1.8–2.0); soft CL wearers (occasional overnight use) 2.2 (CI, 2.0–2.5); daily disposable CL wearers 2.0 (CI, 1.7–2.4); daily disposable CL wearers (occasional overnight use) 4.2 (CI, 3.1–6.6); daily wear silicone hydrogel CL wearers 11.9 (CI, 10.0–14.6); silicone hydrogel CL wearers (occasional overnight use) 5.5 (CI, 4.5–7.2); overnight wear soft CL wearers 19.5 (CI, 14.6–29.5) and in overnight wear of silicone hydrogel 25.4 (CI, 21.2–31.5). Loss of vision occurred in 0.6 per 10 000 wearers. Risk factors included overnight use, poor storage case hygiene, smoking, Internet purchase of CLs, <6 months wear experience, and higher socioeconomic class.

Conclusions: Incidence estimates for soft CL use were similar to those previously reported. New lens types have not reduced the incidence of disease. Overnight use of any CL is associated with a higher risk than daily use.

Financial Disclosure(s): Proprietary or commercial disclosure may be found after the references. *Ophthalmology 2008;115:1655–1662 © 2008 by the American Academy of Ophthalmology.*

Microbial keratitis is a rare but severe complication of contact lens (CL) wear, affecting approximately 5 per 10 000 wearers.[1–4] With a large number of CL wearers worldwide, rare diseases with significant morbidity have considerable public health consequences. Surveillance studies have been used to determine all new cases of disease in populations for New England,[1] Hong Kong,[2] The Netherlands,[3] and Scotland.[4] The denominators in these studies have been estimates of the numbers of wearers in the population derived from community-based telephone surveys. Observations concerning risk factors for the disease have resulted from case control studies carried out in ophthalmic casualty departments.[5–8]

Before 2002, published studies were remarkably consistent in reports of the incidence of CL-related microbial keratitis, with estimates in daily wear soft CL use at 2.7 to 4.1 per 10 000 wearers per year and in overnight CL use at 9.3 to 20.9 per 10 000 wearers per year.[1–4] Since the publication of such studies, CL types and modes of wear have altered. Silicone hydrogel, a highly oxygen-permeable soft CL material type, and daily disposable soft CLs have been introduced. It has been hypothesized that new modalities reduce the risk of disease; however, there are limited population data available to support this.

Contact lens-induced corneal hypoxia may predispose CL wearers to corneal infection because of compromised corneal epithelial integrity,[9] impaired wound healing,[10] and an increased susceptibility of corneal epithelial cells to bacterial binding.[11–13] All CL wear slows corneal epithelial homeostasis by suppressing cell proliferation,[14] impairing cell migration,[15] and reducing the rate of cell exfoliation.[16–18] These effects are reduced but not eliminated with highly oxygen-permeable CLs[14,19]; however, the impact of reduced hypoxia on microbial keratitis is unknown.

Poor CL hygiene[6,20–22] and microbial contamination of the CL storage case[23–25] are frequently implicated in microbial keratitis. The use of daily disposable CLs (CLs worn once and then discarded) avoids the requirement for regular

© 2008 by the American Academy of Ophthalmology
Published by Elsevier Inc.

ISSN 0161-6420/08/$–see front matter
doi:10.1016/j.ophtha.2008.04.002

CL hygiene and the use of a CL storage case and, therefore, may be expected to reduce the risk of CL-related microbial keratitis.[5]

A prospective surveillance study established the incidence of CL-related microbial keratitis in Australia. This study was designed to complement a companion case-control study carried out in London, England, also reported in this issue. The studies share disease definition, severity classification, and CL wearing schedules definition, and used the same hygiene scoring systems so that results can be directly compared.

Methods

There were 2 parts to this study of the incidence of and risk factors for CL-related microbial keratitis. The first identified new cases of microbial keratitis in Australia over a 12-month period (numerator), through a prospective survey of all ophthalmic practitioners in Australia and records review of major ophthalmic centers. Cases were interviewed by telephone to establish risk factor data. The second part established the number of CL wearers in the community (denominator) via a telephone survey. The number of wearers of different CL types was estimated and risk factor information was established through a telephone interview.

Case Ascertainment

Practicing ophthalmologists in Australia were identified through the membership register of the Royal Australian and New Zealand College of Ophthalmologists (Table 1). Practitioners were actively surveyed by e-mail, telephone, fax, or mail at the end of each 2-month reporting period, irrespective of whether eligible cases had been encountered.[26]

New cases of CL-related microbial keratitis between October 1, 2003, and September 30, 2004, were identified. Case detection was augmented by retrospective in-patient and casualty (where available) records and audits at major ophthalmic centers (n = 11).[26]

Optometrists (n = 3288) were similarly contacted at the start of the study and active follow-up was carried out for therapeutically licensed optometrists (n = 173) and members of regional CL societies (n = 444; Table 1).

Informed consent from the case was obtained to allow subsequent collection of clinical data. A telephone interview established demographic and risk factor data. Where possible, interviews were conducted 1 month after the date of the initial event, to limit recall bias; however, delays occurred for cases identified by records audit. Human research ethics approval was obtained from the University of New South Wales Human Research Ethics Committee and 63 regional area health services in Australia.

Study Definitions and Eligibility/Severity Adjudication

The study definitions are common to the companion paper published in this issue[27] and are summarized in Table 2 of the companion paper (available online at http://aaojournal.org).

Cases. Cases were current CL wearers (those who had worn a CL in the previous 4 weeks), aged between 15 and 64 years old and using CLs for the correction of low refractive errors.

Disease Definition and Severity. Microbial keratitis was defined by either a positive corneal culture or a corneal infiltrate and overlying epithelial defect with ≥1 of the following: any part of the lesion within the central 4 mm of the cornea, an anterior chamber response, or pain. Cases were further stratified by severity based on whether visual loss or surgical intervention had occurred and the size and position of the lesion.[27] Where grading was not possible because of a lack of information, cases were apportioned for estimation of incidence according to the known distribution of eligible and ineligible cases. Vision loss was defined as a loss of >2 lines of best-corrected spectacle acuity compared with pre-event acuity (where available), fellow eye acuity (where available), or compared with 20/20. Estimates were also made using a best-corrected spectacle acuity of 6/12.

Table 1. Proportion of Practitioners with Active Follow-up Who Responded at Least Once to the Survey

	Not Practicing	No Contact*	n	Refused	Schedule for Active Follow-up	Participation Rate (≥1 response) [% with complete responses]
Primary source practicing ophthalmologists in Australia						
Australian Ophthalmologists	62	33	661	13 (2.0%)	Email reminder + 2-mthly contact nonresponders	96.2% (636/661) [89.4% (591/661)]
Supplementary source therapeutically licensed optometrists and optometrist who were members of contact lens societies†						
Therapeutically licensed optometrists		23	173	43	E-mail reminder + 6-monthly contact nonresponders	75.1% (130/173)
Contact Lens Society Members	—	35	444	16	E-mail reminder + 6-monthly contact nonresponders	92.8% (428/444)
Total supplementary	—	58	617	59		90.4% (558/617)

*These names were on the lists of practitioners obtained from professional associations; however, a valid postal, telephone, fax, or -email contact could not be established for these practitioners. Therefore, no contact was made during the study.
†Total response rates for optometry with partial active follow-up was 17.9% in Australia.

Contact Lens Wear Modality. Mode of wear was based on wear schedule (daily or overnight wear). Daily wearers were those who habitually removed their CLs for sleep. This was further stratified as daily wear only or occasional overnight users who used lenses primarily on a daily wear basis, but who reported sporadic overnight use of ≤1 night per week. Overnight wearers were those who habitually slept in their CLs (>1 night per week).

Contact Lens Hygiene Compliance. A composite hygiene score, for each subject, was allocated using a weighted scoring system for each of the 4 key areas of CL hygiene: cleaning and disinfection or lens disposal (0–20), storage case replacement (0–4), storage case hygiene (0–8), and hand washing before handling CL (0–8). Scores ranged from 0 for poor to 40 for excellent hygiene.

Other Risk Factors. A wide range of potential risk factors were evaluated, including CL solution type, use of other care or in-eye care products, frequency of CL aftercare, time since last aftercare, lens replacement or disposal frequency, lens age, purchase of CLs via mail order or Internet, CL wear history, indication for CL wear, smoking, hygiene practice after swimming, showering while wearing lenses, and prior history of CL-related complications. Demographic data included age, gender, socioeconomic class, occupation, level of education, and ethnicity.

Population Study

The number of CL wearers in the community was estimated using a national telephone survey of individuals aged 15 to 64 years old in randomly selected households derived from the electronic White Pages and based on the population distribution in Australia.[28] The sample size (32 900 individuals) was estimated based on the likely penetrance of the least common CL type (expected to be 0.13% of the population wearing extended wear silicone hydrogel CL) and for that penetrance to be determined with a precision of ±0.04% indicated that there were 1.75 individuals aged between 15 and 64 years per household; therefore, contact would be required with 18 800 households.

The sample was divided in half, with the first phase surveyed before the beginning of the study, and the second at its conclusion, to avoid the potential for rapidly changing lens types in the community to confound the results. Each study period lasted 4 months. First, households were sent a letter of introduction and up to 4 (phase 1) and 8 (phase 2) attempts were made to make telephone contact. The number of attempted contacts was increased in phase 2 because single person and couple households in the age range of interest were consistently not being reached in Phase 1. Four attempts per household identified 1.97 individuals per household, but 8 attempts yielded 1.88 individuals per household, which was closer to the national estimate for people aged between 15 and 64 years. Based on the actual response rate and the penetrance of silicone hydrogel extended wear, the sample size was adjusted after phase 1 was completed.

A structured questionnaire established the number of all individuals and CL wearers in the household aged between 15 and 64 years. Demographic data were collected and CL wearers were surveyed as described for the cases. Socio-economic status was classified using the Socio-Economic Indexes for Areas based on the 2001 Australian Census.[29]

On completion of the survey, a data quality audit was performed, with 10% of wearers recalled. Weighted κ scores were estimated as measures of agreement between initial and repeated responses.

Data Analysis

Incidence rates were reported as annualized rate per 10 000 wearers for each of the CL wear groups for all presumed microbial keratitis, severe disease, and vision loss.

Univariate analysis initially determined the significance of all possible risk factors using the chi-square and Fisher exact tests for categorical risk factors and the *t* test for those measured on an interval scale. Factors that were significant at $P<0.2$ were considered for multivariate testing using logistic regression. Multivariate analysis ascertained risk factors in the development of any microbial keratitis for daily and overnight users separately. The method of model building comprised initial backward stepwise removal, starting with the most nonsignificant factor and continuing until all variables in the model were significant. Subsequently, each excluded factor was put back into the model, to determine whether its inclusion improved the model. A factor was retained in the final model if either there was a significant improvement in chi-square value, or if confounding occurred. Significant interactions were included in the model. Entry of a variable was dependent on the analyzed sample comprising ≥50% of the available sample. Statistical significance was set at 5%. The strength of association for significant factors was summarized using odds ratios and confidence intervals. The goodness of fit of the final model was assessed using the Hosmer–Lemeshow test and discriminatory ability using the area under the receiver operating characteristic curve.

Results

Case Ascertainment and Severity Adjudication

The overall practitioner response rate was 96.2% (636/661 ophthalmologists; Table 1), and the minimum response rate for any of the 6 reporting periods was 89.4%.

Three hundred forty-four cases of presumed microbial keratitis were reported. Of these, 16 cases were excluded because the onset of disease was outside the surveillance period. A further 7 cases were excluded because they did not meet the inclusion criteria; 5 were keratoconic, 1 aphakic, and 1 wore bandage CLs.

In 46 cases, there was insufficient information to grade the severity. For the purpose of the incidence analysis, these cases were apportioned according to the distribution of eligible/noneligible cases as per the remaining 275 cases. The reviewers graded the remaining 275 cases and 31 (11.3%) did not meet the diagnostic criteria for CL-related microbial keratitis (predictive value positive, 88.7%). Of these 31, 24 were cases of corneal inflammation or sterile/marginal keratitis, 3 were adenoviral keratoconjunctivitis, 1 had herpes simplex keratitis, and 2 had ocular trauma without evidence of an associated infection. Applying this eligible/noneligible case distribution to the 46 cases above gives 41 (89%) eligible cases from this group.

Of the 244 eligible cases with sufficient information to grade severity, there were 34 (13.9%) that had visual acuity reduction of ≥2 lines or required surgical intervention. A further 46 (18.9%) were culture proven without a reduction in vision and 16 (6.6%) were classified as severe keratitis without confirmation of positive culture or loss of visual acuity. There were 61 (25.0%) in the moderate group and 87 (35.7%) were classified with mild disease.

A final set of 285 eligible cases (244 plus 41) was used to calculate the incidence rates for all presumed microbial keratitis. Two subsets were identified, the first composed of 184 cases, which excluded those with mild disease and, second, 39 cases with vision loss. These groups were used to calculate incidence rates for severe disease and for severe disease with visual loss.

Table 2. Penetrance of Contact Lens Wear

Phase	Sample	Contact Lens Wearers	Point Estimate	Penetrance (%, 95% confidence interval)
1	18 348	892	650,565*	4.86 (4.55–5.17)
2	17 566	906	690,192†	5.16 (4.83–5.48)
Combined	35 914	1798	681,424	5.01 (4.78–5.24)

*Based on the census population estimate of 13 381 800.
†Based on census population estimate of 13 568 400.

Population Study

The final sample contacted was composed of 19 754 households and 35 914 individuals interviewed by telephone, including 1798 CL wearers. The data quality was tested in an audit of 176 CL wearers. For all items, the κ scores were >0.8, indicating excellent agreement between the first and second responses.

The overall penetrance of CL wear in the population aged 15 to 64 years was 5.01% (95% confidence interval [CI], 4.78–5.24; Table 2). The distribution of wearers by CL type is shown in Table 3. The penetrance of certain CL types did alter during the course of the study. Both the use of daily disposable CLs and daily silicone hydrogel CLs increased significantly in phase 2, with a reduction in the use of daily wear soft CLs ($P<0.001$). The estimate of penetrance for overnight wear silicone hydrogel CLs was 0.27%, with a precision of ±0.05%.

Incidence Rates

The crude incidence rates are shown in Table 4. A final visual outcome of ≤6/12 occurred in 9.9% of wearers and a reduction of vision of ≥2 lines occurred in 13.9% of wearers.

Multivariate Analysis

The crude risk analysis demonstrated that the risk of microbial keratitis was considerably higher in overnight wear compared with daily or occasional overnight use. Table 5 summarizes the significant variables associated with increased risk of any microbial keratitis.

Table 4. Crude Incidence Rates by Lens Type for All Presumed Microbial Keratitis, "Severe" Keratitis and for Keratitis Causing 2 Lines of Vision Loss

Lens Type	Any Presumed Microbial Keratitis (95% CI)	"Severe" Microbial Keratitis (95% CI)	Microbial Keratitis With >2 Lines Vision Loss (95% CI)
Daily wear RGP	1.2 (1.1–1.5)	1.2 (1.1–1.5)	0 (0.0–0.0)
Pure DW soft	1.9 (1.8–2.0)	1.1 (1.1–1.2)	0.4 (0.4–0.4)
Pure DW DD soft	2.0 (1.7–2.4)	0.5 (0.5–0.6)	0 (0.0–0.0)
Pure DW SH	11.9 (10.0–14.6)	8.0 (6.7–9.8)	1.1 (0.9–1.4)
Occ O/N soft	2.2 (2.0–2.5)	1.8 (1.6–2.0)	0.2 (0.2–0.2)
Occ O/N DD soft	4.2 (3.1–6.6)	2.4 (1.7–3.7)	0 (0.0–0.0)
Occ O/N SH	5.5 (4.5–7.2)	5.3 (4.3–6.9)	1.6 (1.2–2.1)
Overnight wear soft*	19.5 (14.6–29.5)	13.3 (10.0–20.1)	4.0 (2.9–6.6)
Overnight wear SH	25.4 (21.2–31.5)	16.9 (14.1–20.9)	2.8 (2.3–3.5)
Any lens type	4.2 (3.4–5.5)	2.7 (2.2–3.5)	0.6 (0.5–0.7)

CI = confidence interval; DD = daily disposable; Occ O/N = occasional overnight use; Overnight wear = regular overnight use; RGP = rigid gas permeable; "Severe" microbial keratitis = all cases excluding mild; SH = silicone hydrogel.
*Conventional soft contact lenses and planned replacement soft.

Discussion

This paper reports the largest prospective population-based surveillance study of CL-related corneal infection and is the first study to describe the incidence and risks associated with contemporary CL types. Contact lens–related corneal

Table 3. Cases and Controls by Lens Type

	All Controls, n (%)	All Cases, n (%)	Cases of "Severe" Microbial Keratitis, n (%)	Cases with Vision Loss, n (%)
RGP				
Daily wear	140 (7.8)	7 (2.5)	7 (3.8)	0 (0)
Overnight wear	7 (0.4)	0 (0)	0 (0)	0 (0)
Soft				
DD	157 (8.7)	12 (4.2)	3 (1.6)	0 (0)
Occ O/N DD	30 (1.7)	5 (1.8)	3 (1.6)	0 (0)
Daily wear soft*	896 (49.9)	64 (22.5)	39 (21.2)	14 (35.9)
Occ O/N soft*	270 (15.0)	23 (8.1)	18 (9.8)	2 (5.1)
Overnight wear soft*	33 (1.8)	24 (8.4)	15 (8.2)	5 (12.8)
Silicone hydrogel				
Daily wear SH	100 (5.6)	44 (15.8)	30 (16.3)	4 (10.3)
Occasional O/N SH	68 (3.8)	14 (4.9)	14 (7.6)	4 (10.3)
Overnight wear SH	96 (5.4)	92 (32.3)	55 (29.9)	10 (25.6)
Total	1798	285	184	39

DD = daily disposable; Occasional O/N = occasional overnight use; Overnight wear = regular overnight use; RGP = rigid gas permeable; SH = silicone hydrogel.
*Conventional soft contact lenses and planned replacement soft.

Table 5. Multivariate Analysis for All Presumed Microbial Keratitis in Daily and Overnight Wear

	Daily Wear			Overnight Wear		
**Factors**	*P Value*	*Odds Ratio*	*95% CI*	*P Value*	*Odds Ratio*	*95% CI*
Age (y)						
Mid (25–54)		1.00 (referent)			1.00 (referent)	
Young (15–24)	0.86	0.92	0.37–2.28	0.39	1.72	0.50–5.85
Old (55–64)	0.66	1.34	0.36–4.99	0.15	3.78	0.62–23.19
Gender						
Female		1.00 (referent)			1.00 (referent)	
Male	0.38	0.70	0.32–1.55	0.56	1.34	0.49–3.67
Lens material						
Soft hydrogel		1.00 (referent)			1.00 (referent)	
Daily disposable	0.50	1.49	0.47–4.76	—	—	
Silicone hydrogel	0.06	2.62	0.97–7.11	0.76	1.24	0.31–4.97
RGP	0.21	0.27	0.03–2.11	—		
Mode of wear						
Strict daily wear		1.00 (referent)				
Occ O/N 1/month or less	0.79	0.88	0.33–2.30			
Occ O/N at least 1/fortnight	0.01	3.96	1.32–11.86			
Nights of continuous wear						
<6					1.00 (referent)	
≥6				0.14	6.66	0.54–82.33
Storage case hygiene						
Excellent		1.00 (referent)				
Poor	0.001	3.70	1.77–7.75			
Current smoker						
No		1.00 (referent)				
Yes	0.007	2.96	1.34–6.57			
Purchase of CL						
Optometrist		1.00 (referent)				
Internet/mail order	0.03	4.76	1.16–19.58			
Duration of current CL wear (mos)						
>6					1.00 (referent)	
≤6				0.02	4.42	1.31–14.92
Socioeconomic class (SEIFA IQR)						
Low IQR 1–2		1.00 (referent)			1.00 (referent)	
High IQR 3–4	0.01	2.66	1.23–5.76	0.04	2.76	1.08–7.10

CI = confidence interval; CL = contact lens; Occ O/N wear = occasional overnight use; Overnight wear = regular overnight use; SEIFA IQR = Socio-Economic Indexes for Areas interquartile range.
Daily wear model discrimination (area under receiver operating characteristic curve) 79.1%.
Overnight wear model discrimination (area under receiver operating characteristic curve) 73.9%.

infection remains rare, affecting 4.2 per 10 000 (95% CI, 3.4–5.5) wearers in Australia per year. This incidence rate is consistent with those previously reported from the United States,[1] Holland,[3] Hong Kong,[2] and Scotland.[4]

Permanent vision loss after corneal infection occurred in 11% to 13% of cases in studies previously reported.[3,30] The present study reports an annualized incidence in 0.6 per 10 000 wearers (13.9% of cases) who experienced a reduction in best-corrected visual acuity of ≥2 lines. Using a final corrected acuity of 6/12, the rate of vision loss was 9.9%, or 0.4 per 10 000 wearers overall. The highest risk of visual loss occurred with overnight CL use; however, there was no difference in the annual rate of vision loss in soft (4.0 per 10 000) and silicone hydrogel (2.8 per 10 000) use. These estimates are consistent with a recent report of vision loss in overnight silicone hydrogel CL use of 3.6 per 10 000 wearers per year.[31]

Overnight use of CLs, irrespective of material type, continues to be the main risk factor for corneal infection. Overnight use hydrogel CLs had an absolute risk of 19.5 (95% CI, 14.6–29.5) and silicone hydrogel CLs of 25.4 (95% CI, 21.2–31.5) per 10 000 wearers per year. These risks are similar to those previously reported for overnight use of hydrogel CLs[1–3] and a recent report of overnight use of silicone hydrogel CLs.[7,31] There were no significant differences in risk between material types, similar to the finding described by Dart et al.[27] However, of note is the longer continuous duration of overnight wear with silicone hydrogel CL (up to 30 nights) compared with predominantly 6 nights for hydrogel CLs.

The incidence of microbial keratitis associated with daily wear hydrogel CLs is low, (1.9–2.0 per 10 000 wearers per year) and is lower than previous estimates of 2.7 to 6.4 per 10 000 wearers,[1–4,7] which may be expected, because daily wear in this dataset excluded wearers using CLs on an occasional overnight basis. Wear of daily disposable CLs seems to be associated with the lowest risk of severe microbial keratitis. Eliminating the CL storage case may reduce the likelihood of

lens contamination by Gram-negative bacteria, which is associated with more severe disease.[32]

Among daily wearers, the unadjusted incidence of disease was higher with silicone hydrogel CLs than with other daily wear CLs. After controlling for other risk factors, use of silicone hydrogel CLs had a higher but not statistically higher risk of infection than use of hydrogel CL (odds ratio, 2.6; 95% CI, 1.0–7.1). It is possible that factors intrinsic to the material properties of these CLs contribute to the differential risk of infection. However, characteristics of individuals who are early adopters of newly introduced modalities may be important.[33] Conceivably, the risk of microbial keratitis measured with new products may be complicated by the characteristics of the small number of people wearing the latest technology. It might be reasonable to expect that the early adopters of new technology are unique, possibly owing to different demographics, socioeconomic status, compliance, risk-taking behaviors, and lifestyles, or those who may have been fitted with new products after poor success previously. This should be considered in interpretation of epidemiologic studies of CL-related microbial keratitis that focus on the first group of wearers to adopt new products. Such confounding factors could not be addressed with this study design.

Other risk factors in daily wear CL use included sporadic overnight wear and poor storage case hygiene. A strong association with aspects of CL hygiene has been consistently reported.[2,20–22] A higher risk of microbial keratitis among smokers has also been documented previously.[2,8,20] This is the first study to consider the method of CL supply. The higher risk associated with Internet/mail order purchase of lenses is a new finding and this increased risk may be related to CL care attitudes and behaviors. The association between higher socioeconomic class and microbial keratitis in both daily and overnight use was unexpected and remains unexplained.

Risk factors in overnight use of CLs included the duration of current CL wear. A shorter duration of CL use ($<$6 months) was associated with a higher risk of disease, which may suggest some initial adaptive response to CL wear. This is consistent with studies of recovery in CL-induced corneal epithelial thinning and exfoliation rate over time.[19] Similarly, bacterial binding to exfoliated epithelial cells is reduced in wearers with longer duration of wear.[19] These findings would predict that wearers who are able to persist with overnight use are less susceptible survivors.

Limitations of the study include possible underreporting and a reduced capture rate of cases. However, this would be expected to affect all CL types or modalities equally. Also, this might be mitigated by the extremely high response rate (96.2%) among practitioners[1–3] and by the records audit of the major centers. Using 2 sources (surveillance and audit) to detect cases in epidemiologic studies is favored for increased accuracy.[34] Efforts were made to standardize data reporting and case report forms through pilot testing.[35]

This study was designed to evaluate presumed microbial keratitis only. There was some reporting of cases that did not meet the eligibility criteria (11%). These were excluded from the analysis. The use of a clinical case definition has previously been justified in epidemiologic studies[1,3,6,21] and sufficient data were collected in the present study to allow stratification by disease severity.

There is the possibility that bias exists in case detection for newly introduced CL types; however, data capture from multiple sources (treating practitioner, CL wearer, and CL practitioner) likely reduced this occurrence.

The companion study[27] also reported in this issue has mirrored the major results in this study. These include the finding that silicone hydrogel overnight wear has similar risks for microbial keratitis as planned replacement soft overnight wear, and that the risk of developing severe microbial keratitis, with sight loss, is greater for planned replacement soft lenses than with daily disposable soft lenses. A unexpected finding in both studies was that, for the majority of microbial keratitis cases (moderate and mild), there was either no difference between daily disposable and planned replacement soft lenses (Australian study) or a greater risk (UK case control study).

The increased risk of microbial keratitis for daily disposable lenses and some brands of daily disposable lens demonstrated in the UK study, but not this study, may be due to the reduced power of this study to demonstrate differences (12 cases and 116 controls) compared with that of the UK study (84 cases and 482 controls) among daily wear users of daily disposable lenses.

There were differences between the studies for other risk factors. The UK study showed that longer periods of daily wear, poor hand hygiene, hypermetropia, younger age, and male gender were risk factors, whereas this study showed poor CL case hygiene, smoking, Internet supply of lenses, and higher socioeconomic class to be risk factors. These differences probably relate to differences in CL practice between the 2 countries and differences in study power owing to differences in market penetrance of the different modalities.

These 2 new studies, taken together, represent the largest prospective, well-designed, epidemiologic studies of this problem and provide definitive data to help the CL user and CL practitioner make informed choices about CL types and wear schedules. They also provide the CL industry with information that might be expected to lead to the development of safer lens types.

In summary, CL-related microbial keratitis remains an issue of significant public health importance. Although the risk to the individual is low, the population affected by this condition is young, healthy, and of working age, with a low risk of infection in the absence of CL wear. Affected individuals may experience vision loss, a period of hospital admission, multiple outpatient visits, and significant pain and discomfort. This paper reports the first prospective surveillance study to establish risks with newly introduced CL modalities. Overnight wear remains the major risk factor for developing microbial keratitis, irrespective of material type. Other risk factors include poor storage case hygiene, smoking, method of CL purchase, and socioeconomic class. Users of overnight wear CLs have an increased risk for developing microbial keratitis during their early wear experience. The risk of infection associated with CL use needs to be

considered in the context of informed choice and risk–benefit, including the risks associated with refractive surgery and the benefits of the cosmetic, sporting, and other advantages of CL wear.

References

1. Poggio EC, Glynn RJ, Schein OD, et al. The incidence of ulcerative keratitis among users of daily-wear and extended-wear soft contact lenses. N Engl J Med 1989;321:779–83.
2. Lam DS, Houang E, Fan DS, et al. Incidence and risk factors for microbial keratitis in Hong Kong: comparison with Europe and North America. Eye 2002;16:608–18.
3. Cheng KH, Leung SL, Hoekman HW, et al. Incidence of contact-lens-associated microbial keratitis and its related morbidity. Lancet 1999;354:181–5.
4. Seal DV, Kirkness CM, Bennett HG, et al. Population-based cohort study of microbial keratitis in Scotland: incidence and features. Cont Lens Anterior Eye 1999;22:49–57.
5. Dart JKG, Stapleton F, Minassian D. Contact lenses and keratitis [letter]. Lancet 1991;338:1146–7.
6. Stapleton F, Dart JK, Minassian D. Risk factors with contact lens related suppurative keratitis. CLAO J 1993;19:204–10.
7. Morgan PB, Efron N, Hill EA, et al. Incidence of keratitis of varying severity among contact lens wearers. Br J Ophthalmol 2005;89:430–6.
8. Morgan PB, Efron N, Brennan NA, et al. Risk factors for the development of corneal infiltrative events associated with contact lens wear. Invest Ophthalmol Vis Sci 2005;46:3136–43.
9. Madigan MC, Holden BA. Reduced epithelial adhesion after extended contact lens wear correlates with reduced hemidesmosome density in cat cornea. Invest Ophthalmol Vis Sci 1992;33:314–23.
10. Mauger TF, Hill RM. Corneal epithelial healing under contact lenses: quantitative analysis in the rabbit. Acta Ophthalmol (Copenh) 1992;70:361–5.
11. Imayasu M, Petroll WM, Jester JV, et al. The relation between contact lens oxygen transmissibility and binding of *Pseudomonas aeruginosa* to the cornea after overnight wear. Ophthalmology 1994;102:371–88.
12. Cavanagh HD, Ladage PM, Yamamoto K, et al. Effects of daily and overnight wear of hyper-oxygen transmissible rigid and silicone hydrogel lenses on bacterial binding to the corneal epithelium: 13-month clinical trials. Eye Contact Lens 2003;29(suppl):S14–16.
13. Latkovic S, Nilsson SE. The effect of high and low Dk/L soft contact lenses on the glycocalyx layer of the corneal epithelium and on the membrane associated receptors for lectins. CLAO J 1997;23:185–91.
14. Ladage PM, Ren DH, Petroll WM, et al. Effects of eyelid closure and disposable and silicone hydrogel extended contact lens wear on rabbit corneal epithelial proliferation. Invest Ophthalmol Vis Sci 2003;44:1843–9.
15. Ladage PM, Jester JV, Petroll WM, et al. Vertical movement of epithelial basal cells towards the corneal surface during use of extended-wear contact lenses. Invest Ophthalmol Vis Sci 2003;44:1056–63.
16. Ladage PM, Yamamoto K, Ren DH, et al. Effects of rigid and soft contact lens daily wear on corneal epithelium, tear lactate dehydrogenase, and bacterial binding to exfoliated epithelial cells. Ophthalmology 2001;108:1279–88.
17. Ren DH, Petroll WM, Jester JV, et al. The relationship between contact lens oxygen permeability and binding of *Pseudomonas aeruginosa* to human corneal epithelial cells after overnight and extended wear. CLAO J 1999;25:80–100.
18. Stapleton F, Kasses S, Bolis S, Keay L. Short term wear of high Dk soft contact lenses does not alter corneal epithelial cell size or viability. Br J Ophthalmol 2001;85:143–6.
19. Ren DH, Yamamoto K, Ladage PM, et al. Adaptive effects of 30-night wear of hyper-O(2) transmissible contact lenses on bacterial binding and corneal epithelium: a 1-year clinical trial. Ophthalmology 2002;109:27–39.
20. Schein OD, Glynn RJ, Poggio EC, et al, Microbial Keratitis Study Group. The relative risk of ulcerative keratitis among users of daily-wear and extended-wear soft contact lenses: a case-control study. N Engl J Med 1989;321:773–8.
21. Dart JK, Stapleton F, Minassian D. Contact lenses and other risk factors in microbial keratitis. Lancet 1991;338:650–3.
22. Radford CF, Minassian DC, Dart JK. Disposable contact lens use as a risk factor for microbial keratitis. Br J Ophthalmol 1998;82:1272–5.
23. Mayo MS, Schlitzer RL, Ward MA, et al. Association of *Pseudomonas* and *Serratia* corneal ulcers with use of contaminated solutions. J Clin Microbiol 1987;25:1398–400.
24. Wilson LA, Sawant AD, Simmons RB, Ahearn DG. Microbial contamination of contact lens storage cases and solutions. Am J Ophthalmol 1990;110:193–8.
25. McLaughlin-Borlace L, Stapleton F, Matheson M, Dart JK. Bacterial biofilm on contact lenses and lens storage cases in wearers with microbial keratitis. J Appl Microbiol 1998;84:827–38.
26. Keay L, Edwards K, Brian G, Stapleton F. Surveillance of contact lens related microbial keratitis in Australia and New Zealand: multi-source case-capture and cost-effectiveness. Ophthalmic Epidemiol 2007;14:343–50.
27. Dart JKG, Radford CF, Minassian D, et al. Risk factors for microbial keratitis with contemporary contact lenses: a case control study. Ophthalmology 2008;115:1647–54.
28. Australian Bureau of Statistics. 1301.2 Year Book Australia 2002. Section 6: estimated resident population by age and sex. Available at: www.ausstats.abs.gov.au/ausstats/. Accessed March 18, 2008.
29. Australian Bureau of Statistics. 2001 Census of Population and Housing-SEIFA 2001. Available at: www.abs.gov.au/websitedbs/. Accessed March 11, 2008.
30. Nilsson SE, Montan PG. The annualized incidence of contact lens induced keratitis in Sweden and its relation to lens type and wear schedule: results of a 3-month prospective study. CLAO J 1994;20:225–30.
31. Schein OD, McNally JJ, Katz J, et al. The incidence of microbial keratitis among wearers of a 30-day silicone hydrogel extended-wear contact lens. Ophthalmology 2005;112:2172–9.
32. Keay L, Edwards K, Naduvilath T, et al. Factors affecting the morbidity of contact lens-related microbial keratitis: a population study. Invest Ophthalmol Vis Sci 2006;47:4302–8.
33. Keay L, Radford C, Dart JK, et al. Perspective on 15 years of research: reduced risk of microbial keratitis with frequent-replacement contact lenses. Eye Contact Lens 2007;33:167–8.
34. McCarty DJ, Tull ES, Moy CS, et al. Ascertainment corrected rates: applications of capture-recapture methods. Int J Epidemiol 1993;22:559–65.
35. Keay L, Edwards K, Brian G, et al. Evaluation of surveillance methods for an epidemiological study of contact lens related microbial keratitis. Clin Experiment Ophthalmol 2004;32:349–53.

Footnotes and Financial Disclosures

Originally received: November 6, 2007.
Final revision: March 24, 2008.
Accepted: April 1, 2008.
Available online: June 5, 2008. Manuscript no. 2007-1439.

[1] Institute for Eye Research, Sydney, Australia.

[2] School of Optometry and Vision Science, University of New South Wales, Sydney, Australia.

[3] Vision CRC, Sydney, Australia.

[4] Moorfields Eye Hospital, London, United Kingdom.

[5] Institute of Ophthalmology, University College, London.

[6] International Centre for Eyecare Education, Sydney, Australia.

Financial Disclosure(s):
There are no competing interests for any of the authors. The Vision CRC receives a royalty on the sales of silicone hydrogel lenses.

Supported by the Institute for Eye Research (FS, TN), CIBA Vision USA (all authors), the University of New South Wales (FS), the Vision Cooperative Research Centre (KE, LK), the National Health and Medical Research Council (LK). The authors were responsible for design and conduct of the study; collection, management, analysis, and interpretation of the data; and preparation, review, or approval of the manuscript. A draft of the manuscript was provided to the Institute for Eye Research and CIBA Vision before publication, and modifications were limited to clarification and editorial changes.

Correspondence:
Prof Fiona Stapleton, School of Optometry and Vision Science, University of New South Wales, Sydney, New South Wales, 2052, Australia. E-mail: F.Stapleton@unsw.edu.au.
